# Maternal trans-vaccenic acid shapes neonatal T cell development and early-life immune imprinting

**DOI:** 10.1126/science.aea4041

**Published:** 2026-06-18

**Authors:** Hao Fan, Zhong Zheng, Kaitlyn Oliphant, Jiacheng Li, Ryan Mack, Cheng-Wei Ju, Brandon Trandai, Jiayi Tu, Freya Q. Zhang, Rukang Zhang, Zhicheng Xie, Chunzhao Yin, Chufan Cai, Megan S. Kennedy, Tess McNeely, Candace Cham, Hardik Shah, Lei Dong, Rui Su, Camilia R. Martin, Brian T. Layden, Robert B. Hamanaka, Gökhan M. Mutlu, Eugene B. Chang, Jiwang Zhang, Hongbo Chi, Erika C. Claud, Chuan He, Jing Chen

**Affiliations:** 1 Department of Medicine, The University of Chicago; Chicago, 60637, USA.; 2 Department of Chemistry, The University of Chicago; Chicago, 60637, USA.; 3 Department of Pediatrics, The University of Chicago; Chicago, 60637, USA.; 4 Department of Cancer Biology, Loyola University Chicago; Maywood, 60153, USA; 5 Medical Scientist Training Program, Pritzker School of Medicine, The University of Chicago, Chicago, 60637, USA; 6 Quantitative Biomedical Research Center, University of Texas Southwestern Medical Center; Dallas, 75390, USA.; 7 Department of Systems Biology, Beckman Research Institute of City of Hope; Duarte, 91010, USA.; 8 Division of Neonatology, Weill Cornell Medicine; New York, 10065, USA.; 9 Department of Medicine, University of Illinois Chicago; Chicago, 60612, USA.; 10 Department of Immunology, St Jude Children’s Research Hospital; Memphis, 38105, USA.

## Abstract

How maternal nutrition influences neonatal immune development and imprinting through breastfeeding remains largely unclear. We report that maternal supplementation with trans-vaccenic acid (TVA), the predominant naturally occurring trans-fatty acid in human breast milk (HBM), promoted neonatal T cell development in mice. Neonates fed by mothers on a TVA-enriched diet showed an expanded naïve CD4^+^ T cell population and enhanced adaptive immunity against infection. TVA reprogrammed neonatal naïve CD4^+^ T cells through a G protein-coupled receptor (GPR43)-CCCTC-binding factor axis and promoted Th1-skewing by cooperating with the transcription factor TBX21. Early-life exposure to maternal TVA *via* breastfeeding supported long-lasting antiviral immunity in adulthood. Our findings establish the multifaceted benefits of maternal nutrition and breastfeeding *via* TVA in promoting infant immune homeostasis and protective immunity.

Early-life nutritional exposures can shape physiology and disease risk across the lifespan, yet the mechanisms by which maternal diet influences neonatal immune development remain poorly understood. Maternal nutrition is a critical determinant of human breast milk (HBM) composition, which is pivotal for breastfeeding to help build the infant’s immune system and lower the risk of infections and dysfunctional organ development (1–4). HBM contains nutritional components such as fatty acids, carbohydrates, and vitamins, as well as immunomodulatory substances, including infection-protecting antibodies, anti-inflammatory cytokines, and bacteria for the establishment of healthy microbial gut colonization for effective immunological responses against infection and inflammation in newborns(1, 5–8). Therefore, the benefits of breastfeeding are generally attributed to the combined action of multiple components, raising the question of whether a single dietary molecule in breast milk can directly shape neonatal immune development and long-term immune imprinting.

Maternal intake of dietary fatty acids directly impacts the composition and levels of fatty acids in HBM, which can be critical for immune development in infants by playing a nutritional role and influencing the gut microbiome(9, 10). However, it remains unclear whether a single diet-derived fatty acid delivered through breastfeeding can independently instruct immune development in newborns. TVA is the predominant naturally occurring trans-fatty acid in HBM and is derived exclusively from maternal diet because the human body cannot produce TVA(11–13). TVA in humans is mainly from ruminant-derived food, including beef, lamb, milk, and butter, but naturally less than 20% of dietary TVA is metabolized by the human body(12–15). We recently reported that the circulating TVA enhances adult CD8^+^ effector T cell function and anti-tumor immunity(16). TVA can function as an immunomodulator and inactivate CD8^+^ T cell-surface receptor GPR43, an immunomodulatory G protein-coupled receptor (GPCR), triggering activation of the cyclic adenosine monophosphate (cAMP)-protein kinase A (PKA)-cAMP-response element-binding protein (CREB) axis to reprogram adult CD8^+^ T cells. We thus asked whether maternal TVA as a single HBM nutrient *via* breastfeeding could shape neonatal immune development and immunity.

## Maternal TVA reshapes neonatal T cell development *via* breastfeeding

We found that TVA levels in HBM samples from mothers correlated to serum TVA levels from their preterm infants (Fig. 1A). To test the effects of maternal dietary TVA on neonatal immune development, lactating mouse dams were fed diets supplemented with 0.1% or 1% TVA starting in pregnancy (Fig. 1B). Both TVA-supplemented groups produced milk with comparable TVA levels, whereas no TVA was detected in milk from dams on a control diet (Fig. 1C). Pups fed TVA-milk showed time-dependent serum accumulation of TVA until weaning (3 weeks post-birth; on TVA-enriched diet afterwards), whereas control pups had no detectable serum TVA (Fig. 1D). Pups on TVA-milk (TVA-pups) or control milk had comparable body weights (BWs) (fig. S1A), suggesting that TVA in milk did not affect neonatal growth.

Spleen size was increased in TVA-pups compared to controls at 2-3 weeks but became comparable by 5 weeks (Fig. 1E). At 2 weeks, TVA-pups had elevated numbers of total splenocytes, CD4^+^ and CD8^+^ T cells, B cells, neutrophils, dendritic cells (DCs), and monocytes in spleens (fig. S1B), with only increased frequency of CD4^+^ T cells (fig. S1C). In contrast, by 5 weeks, total splenocytes and most immune cell numbers were comparable (fig. S1D), with increased CD8^+^ T cell frequency and reduced percentages of B cells and DCs (fig. S1E). Further analysis at 2 weeks revealed increased CD4^+^ and CD8^+^ T cell numbers (fig. S1F–G) and elevated naïve CD4^+^ T cell frequency (fig. S1H) in spleens of TVA-pups, whereas other T cell subsets were unchanged (fig. S1H–K) except for increased expression levels of the T cell activation marker, CD69, on CD8^+^ T cells (fig. S1K).

TVA-pups (2 weeks) also had increased thymus weights (fig. S1L), with reduced frequency of CD4/CD8 double-negative (DN) precursors but increased frequency of later stage CD4/CD8 double-positive (DP) thymocytes, whereas thymotic percentages of both CD4^+^ (4SP) and CD8^+^ (8SP) cells were comparable between TVA-pups and controls (fig. S1M). We did not observe differences between TVA-pups and controls (2 weeks) in the frequencies of CD4^+^ and CD8^+^ T cells, regulatory T cells, naïve T cells, or CD69^+^ T cells within the lung immune microenvironment (Fig. S1N–Q).

We performed single-nuclei (sn) multi-omics, including sn-RNA-seq and sn-ATAC-seq, to characterize transcriptional profiles and chromatin accessibility of splenic cells of TVA-pups and controls (2 weeks; Fig. 1F, Data S1–2). We identified 20 distinct clustered populations (Fig. 1F, S2A, Data S3). TVA-pups exhibited increased frequencies of T cell subsets, including multiple CD4^+^ and CD8^+^ populations that mapped to distinct differentiation states. Naïve CD4^+^ (cluster C3) and naïve CD8^+^ (C4) cells expressed lymphoid-homing and quiescence-associated genes (*Sell, Ccr7, Tcf7, Lef1*, and *Il7r*) without a cytotoxic module. CD4 effector cells (C9) upregulated a Th1-like activation module, including the type 1 cytokine *Ifng*, the Th1 lineage factor *Tbx21*, the trafficking receptor *Cxcr3*, and the effector molecule *Gzmk*, together with low levels of exhaustion-associated genes (*Pdcd1/Tox/Lag3*), consistent with recently activated Th1-skewed cells. CD8 clusters further resolved into *Bcl11b*^+^ naïve-like/precursor state poised for differentiation cells (C6), ICOS^+^ early-activated/progenitor-exhausted cells (C10; *Icos, Pdcd1, Tox, Lag3*, minimal *Gzmb*), and terminal effector cells (C15; *Gzmb, Prf1, Zeb2, Cx3cr1, Ifng, Tbx21*). The naïve CD4^+^ subset (C3) represented the most expanded T cell population in the TVA group. Moreover, γδ T cells (C20), together with mature follicular B cells (C5) and classical dendritic cells (C18), all critical for T cell–dependent adaptive immunity, were also expanded compared with controls (Fig. 1G).

In splenic T cells isolated from TVA-pups compared to controls, we identified increased promoter accessibility scores (promoter sums), reflecting greater chromatin accessibility at the promoters of representative genes related to T cell maturation and function (e.g. *Il2rb*, *Tnfrsf9*, *Ifng*, *Gzmb*, and *Icos*), proliferation and survival (e.g. *Bcl2*, *Bcl11b*, and *Cd28*), and migration and trafficking (e.g. *Ccl5*, *Cxcr6*, and *Ccr8*) (Fig. 1H). The promoter sums of *Gpr43* (*Ffar2*), which was identified as a TVA target in adult CD8^+^ effector T cells(16), were increased in clusters of T cells that are expanded in TVA-pups compared to controls, including naïve CD4^+^ T cells (C3), naïve CD8^+^ T cells (C4), CD4^+^ effector T cells (C9), CD8^+^ T cells (C10), and CD8^+^ effector T cells (C15) (Fig. 1I). Transcription factor (TF) motifs of CREB and TBX21 (T-bet) were increased in TVA-expanded neonatal naïve CD4^+^ T cells (C3) (fig. S2B–C). We concluded that TVA-milk primarily promoted neonatal T cell development, especially in the naïve CD4^+^ T cell compartment.

## TVA-milk reprograms neonatal CD4^+^ naïve T cells through a GPR43-CTCF axis

To determine the role of the cell-surface receptor GPR43(16), we examined the effects of TVA on neonatal mice which had naïve CD4^+^ T cells that were deficient in *Gpr43* (*Gpr43*^−/−^) (Fig. 2A, *left*). CD4^+^ T cells isolated from neonatal mice fed on TVA-milk showed increased Th1 polarization *ex vivo* (assessed by %T-bet^+^IFN-γ^+^ in CD4^+^ T cells), activation (assessed by CD69 expression), and cell expansion (Fig. 2A *right*, S2D) but had minimal effects on Th2 and Th17 polarization (fig. S2E–F). CD4^+^ T cells from *Gpr43*^−/−^ pups on control milk showed elevated Th1 polarization, activation, and cell expansion compared to cells from control pups (Fig. 2A right, S2D). This suggested that GPR43 might have an immunosuppressive role in neonatal CD4^+^ T cells. In contrast, TVA-milk had minimal effects on neonatal CD4^+^ T cells in *Gpr43*^−/−^ pups (Fig. 2A *right*, S2D).

In addition, we compared the effects of the three most abundant unsaturated fatty acids in milk: oleic acid, linoleic acid, and palmitoleic acid; and the three most abundant trans fatty acids: TVA, elaidic acid, and conjugated linoleic acid. Only TVA increased the frequency of T-bet^+^IFN-γ^+^ CD4^+^ T cells (Fig. S3A). This effect was completely absent in cells from *Gpr43*^−/−^ pups (Fig. S3B). Consistent with our previous finding that TVA inactivates GPR43 to increase cAMP production and PKA activity(16), treatment with cell permeable 8-Bmoro-cAMP and PKA inhibitor resulted in increased and decreased Th1 polarization of neonatal naïve CD4^+^ T cells, respectively, and both abolished the effects of TVA (fig. S3C). However, treatment with CREB inhibitor had minimal effects on Th1 polarization of neonatal naïve CD4^+^ T cells, nor did it affect TVA-enhanced Th1 polarization (fig. S3C). These data suggested that TVA reprogrammed neonatal naïve CD4^+^ T cells through the GPR43-cAMP-PKA axis, which, however, was CREB-independent.

To identify the TFs responsible for the effects of TVA on neonatal naïve CD4^+^ T cells, we focused on 4 subclusters with distinct TVA-dependent patterns of TF activity in the splenic neonatal naïve CD4^+^ T cell population (Fig. 2B–C). CTCF in subcluster 1 represented the only TF with increased activity (Fig. 2D, fig. S3D), whereas minimal changes of CREB were detected (fig. S3E). CTCF (CCCTC-binding factor) is a zinc finger TF that regulates three-dimensional genome organization and prevents improper interactions between enhancers and promoters(17). CTCF cooperates with TBX21 (T-bet), which is critical for Th1 polarization to ensure proper gene expression in Th1 cells, including *Ifng* (18). The promoters of *Tbx21* and *Ifng* showed higher accessibility in subcluster 1 compared with the other subclusters (fig. S3F). We found that knockdown of *Ctcf*, but not *Creb1* (fig. S3G), abolished Th1 polarization and TBX21 (T-bet) activation enhanced by either TVA or 8-Bmoro-cAMP in neonatal naïve CD4^+^ T cells (Fig. 2E). Moreover, treatment with TVA enhanced CTCF TF activity assessed by increased DNA binding ability in neonatal naïve CD4^+^ T cells, which was not further enhanced by 8-Bmoro-cAMP and could be abolished by treatment with a PKA inhibitor (Fig. 2F, fig. S3H). Since the promoter sums of *Ctcf* were not altered in subclusters of naïve CD4^+^ T cells (fig. S3I), we concluded that TVA reprogrammed neonatal naïve CD4^+^ T cells through a GPR43-cAMP-PKA-CTCF axis, likely by enhancing CTCF TF activity but not its expression.

## TVA supplementation does not broadly alter milk composition or the early-life microbiome

To determine whether maternal TVA broadly changed immunologically relevant milk components, we measured representative milk immune factors. The levels of IgA, lactoferrin, and cytokines, including IL-1b and IL-7, in milk from dams on the TVA diet were comparable to those in milk from control dams (fig. S4A–D). Comprehensive lipid (fatty acids) profiling analysis of milk from the TVA and CON groups revealed that TVA was the only fatty acid that differed between the two groups (fig. S4E, Data S4). In addition, the diversity and patterns of microbial distribution in mouse milk varied widely without a clear trend among samples from different mice in the same group of dams, suggesting that feeding TVA-enriched diet to mouse dams did not alter the milk microbiome substantially (fig. S4F–I). Analysis of the fecal microbiome in 2-week-old pups by 16S rRNA sequencing revealed no substantial differences in microbial diversity between TVA and CON groups. Instead, litter (cage effect) was the dominant source of microbiome variation, with no clear separation between cohorts (Fig. S5A–C). We performed Low-input Multiple Methylation Sequencing (LIME-seq) on 2-week-old pups’ fecal RNA to assess microbial abundance and methylation signatures(19). Consistent with the 16S data, LIME-seq showed highly similar microbial compositions between groups (Fig. S5D–E), and differential analysis of microbiome-associated methylation ratios, reflecting microbial metabolic activity, did not identify robust group-specific differences (Fig. S5F). These results were consistent with the early-life gut microbiome being unaffected by dietary TVA, and that observed variability was primarily driven by litter-associated effects rather than diet.

## TVA-milk programs neonatal T cells for anti-viral responses

To determine the functional effect of maternal TVA on neonatal immunity, we used a neonatal mouse model of lung infection with the H1N1 influenza A virus A/Puerto Rico/8/1934 (PR8)(20) *via* intranasal administration (Fig. 3A). Although TVA- and control pups at 10 days showed comparable BWs (fig. S6A), TVA-pups had enhanced survival (Fig. 3B) and reduced clinical score (fig. S6B–C) following influenza A infection. Moreover, pathologic analysis of lung tissues from control pups showed that influenza A virus caused lung epithelial damage (fig. S6D, *upper*, quantitative results shown in Fig. 3C, *left*) with reduced normal epithelium (fig. S6D, *lower*, quantitative results shown in Fig. 3C, *right*), compared to pups without viral treatment. TVA-pups showed reduced lung damage and increased normal epithelium, compared to controls with viral infection (fig. S6D, quantitative results shown in Fig. 3C).

Further analysis of lung tissues revealed that, compared to controls, TVA-pups had reduced viral loads in lungs as early as Day 7 and Day 10 post-infection (Fig. 3D). Although the frequency of lung-infiltrating CD4^+^ T cells (gating strategies in fig. S7A) was not altered in TVA-pups (fig. S7B), these cells had elevated CD69 and CD44 levels (Fig. 3E, *left*, fig. S7C) on Day 7 post-infection. In addition, the frequency of influenza-specific CD4^+^ T cells accessed as CD11a^+^/CD49d^+^- (fig. S7D) and NP_331-325_^+^ (epitope of viral N protein)-specific cells (Fig. 3E *right*, S7E) were also elevated in TVA-pups on Day 7 but reduced on Day 10 post-infection, whereas these populations peaked on Day 10 post-infection in controls. Similar results were obtained for lung-infiltrating CD8^+^ T cells (fig. S7F, Fig. 3F, *left*). The frequency of influenza-specific (PA_224-233_^+^) CD8^+^ T cells was elevated on Day 7 but reduced in TVA-pups on Day 10 post-infection, compared to this population in controls that was increased only on Day 10 post-infection (Fig. 3F *right*, S7G). *Ex vivo* activation of lung CD4^+^ and CD8^+^ T cells following viral infection revealed that, compared with controls, TVA-pups exhibited an increased expression of IFN-γ, T-bet^+^, and TNF-α by CD4^+^ T-cell subsets. The frequencies of IFN-γ^+^ and TNF-α^+^ cells were also markedly increased within the CD8^+^ T-cell compartment (Fig. S8A). T_reg_ cells in the lung and spleen following influenza A virus infection showed no differences between TVA-pups and controls (Fig. S8B).

Most of the populations of lung-infiltrating B cells, NK cells, macrophages, DCs, neutrophils, and monocytes (fig. S8C–H) were not altered in TVA-pups compared to controls. Only populations of lung-infiltrating B cells and NK cells were increased or reduced in TVA-pups on Day 10 post-infection, respectively. TVA-pups showed reduced inflammation assessed by decreased IL-1b in lungs (Fig. 3G) and TNF-a levels in lungs and serum (fig. S8I–J). When TVA exposure was restricted to first-generation pups (Fig. S9A), the spleen development (Fig. S9B) and influenza A virus response results of pups (Fig. S9C) remained consistent with our previous findings.

We concluded that TVA-milk enhanced the readiness of neonatal T cells, leading to an early but more effective response to viral infection and inflammation in lungs to improve the survival of virus-infected pups.

## TVA-milk enacts a shift to Th1-skewing immunity against viral infection

We performed snMulti-Omics analysis of lung tissue cells of pups that were sacrificed on Day 7 post-viral infection (fig. S10A, Data S5–6). Results of snRNA-seq identified 20 distinct clusters (Fig. 3H, S10B, Data S7). Upon viral infection, pups on TVA-milk had increased lung-infiltrating populations of diverse T cell subsets including CD8^+^ effector T cells and Th1-like T cells, but reduced lung-infiltrating Th2-like T cells compared to controls (Fig. 3I). These results suggested that maternal TVA through breastfeeding implemented a shift from Th2-biased neonatal immunity to Th1-skewing, which enhanced recruitment of lung infiltrating Th1 cell subset to help clear viral loads more effectively in infected lungs.

Lung-infiltrating cell clusters with increased populations had higher *Gpr43* transcript levels (fig. S10C–D). In addition, snATAC-seq analysis revealed a total of 15 clusters (fig. S10E), of which 4 clusters had increased cell populations in lung tissues from TVA-pups with viral infection. These clusters were identified as Th1 cells (C1) and CD8^+^ effector T cells (C4, C7, and C9 (fig. S10F), and which had indications of increased TF activity of TBX21 (T-bet) (fig. S10G). The activity of CREB was increased in lung-infiltrating CD8^+^ effector T cell populations, but not Th1 cells, of TVA-pups compared to controls (fig. S10H), consistent with our finding that TVA-milk functioned through GPR43-dependent activation of CTCF.

To examine the role of TBX21 and Th1-skewing shift in effects of maternal TVA on neonatal immunity, we used Tbx21 knockout (*Tbx21*^−/−^) mice that cannot effectively develop Th1 cells (Fig. 3J). *Tbx21* gene deficiency abolished anti-viral effects of TVA-milk, leading to comparable survival and clinical score between TVA-pups and controls (Fig. 3K, fig. S11A–B). Similar results were obtained using TCR-α knockout (KO) mice deficient of αβ T cells (fig. S11C–E), and CD4 KO mice (fig. S11F–H). To assess the role of GPR43, we used *Gpr43* whole-body KO (*Gpr43*^−/−^) and T cell-specific *Gpr43* KO (*Gpr43*^fl/fl^CD4^Cre^) mice. TVA treatment improved clinical scores and survival in littermate controls but not in *Gpr43*^−/−^ (fig. S11I–K) or *Gpr43*^fl/fl^CD4^Cre^ pups (Fig. 3L, S11L). These results were consistent with GPR43 expression on CD4^+^ T cells mediating the antiviral protective effects of TVA in neonates.

We employed an adoptive transfer approach (fig. 4A *left*, S12A) to compare CD4^+^ T cells from TVA-pups and controls. Endogenous CD4^+^ T cells were depleted in CD45.2 recipients using an anti-CD4 antibody. Neonatal naïve CD4^+^ T cells from 2-week-old pups were co-transferred at a 1:1 ratio: CD45.1 cells from controls and CD45.1/2 cells from TVA-pups (ratio confirmed by flow cytometry, fig. S12B). Recipient CD45.2 mice, maintained on a control diet, were challenged with influenza A virus. Seven days post-infection, CD45.1/2 CD4^+^ T cells (derived from TVA-pups) outcompeted CD45.1 CD4^+^ T cells (from controls) in the spleen (fig. 4A *right*, S12C *upper*). This advantage was even more pronounced among lung-infiltrating Th1 cells (fig. 4A *middle*, S12C *lower*), where the CD45.1/2:CD45.1 ratio was higher in the lung than in the spleen (fig. S12D). These data indicated that TVA-programmed neonatal naïve CD4^+^ T cells possessed a cell-intrinsic advantage in survival, expansion, and Th1 differentiation upon viral challenge.

We established a Th1-dependent infection model using *Salmonella enterica serovar Typhimurium* (*S. Typhimurium*), in which bacterial burden is assessed in the spleen, liver, and lung. Breeders were fed CON or TVA diets, and their 2-week-old pups were challenged with *S. Typhimurium* (Fig. 4B *left*). By day 3 post-challenge, infection disrupted normal body-weight gain in CON pups, whereas TVA mitigated this effect (fig. S13A). TVA-pups showed reduced bacterial colonization in spleen and liver (Fig. 4B *middle*). Overall CD4^+^ frequencies among CD45^+^ leukocytes were comparable between groups (fig. S13B *left*), but TVA enhanced Th1 polarization, increasing T-bet^+^CD4^+^ (Fig. 4B *right*) and IFN-γ^+^CD4^+^ T cells (fig. S13B *right*), and IFN-γ^+^CD8^+^ T cells (fig. S13C). Other splenic immune populations were unchanged, apart from neutrophils, which were reduced in TVA-pups, consistent with decreased bacterial burden (fig. S13D).

## TVA in HBM inversely correlates with the incidence of preterm bronchopulmonary dysplasia

We examined the relationship between TVA level and cytokine profile in the blood of mouse pups and human infants. Serum TVA levels of TVA-pups (2 weeks) positively correlated with IL-2, IL-22, and CXCL9 levels, all of which are related to T cell functions, but inversely correlated with IL-4 (fig. S14A, Data S8). Similar results were obtained using blood spot samples from preterm infants (Fig. 4C, Data S9). Infant blood TVA levels correlated positively with IL-2, negatively with IL-4, and with an increased IFN-γ/IL-4 ratio (Fig. 4C, *left*, *middle*, and *right*). Given that IL-2 is important for naïve T cell expansion and the IFN-γ/IL-4 ratio is a widely used readout of Th1/Th2 balance(21), this finding was consistent with TVA-milk preferentially promoting T cell expansion and a shift toward Th1 neonatal immunity. Moreover, to directly test the effect of TVA on human neonatal naïve CD4^+^ T cells, we isolated naïve CD4^+^ T cells from human umbilical cord blood and treated them with TVA *in vitro*. Consistent with our mouse data, TVA treatment enhanced Th1 polarization in all three independent cord blood samples (Fig. 4D, S14B–C).

Preterm infants have enfeebled Th1 immune responses and thus are vulnerable to infections, which may cause chronic inflammation, leading to disrupted organ development with an increased risk of diseases such as bronchopulmonary dysplasia (BPD), which is strongly associated with lung inflammation(22–24). We used a mass spectrometry-based fatty acid profiling approach to examine the link between human milk fatty acids and preterm-associated inflammatory BPD (Fig. 4E, *left*). We found that TVA levels in HBM samples from mothers as the first in rank, inversely correlated with the incidence of BPD for their preterm infants (Fig. 4E, *right*, Data S10–11). Moreover, TVA levels in the blood spot samples from a group of preterm infants inversely correlated with BPD incidence (Fig. 4F, Data S12). These results together suggested a link between TVA in breastmilk and neonatal T cell function lowering risk of preterm inflammatory BPD.

## Early-life exposure to TVA *via* breastfeeding impacts immune imprinting

To distinguish prenatal and postnatal TVA exposure *via* breastfeeding, we used a mouse-fostering strategy(25) in which pups born to C57BL/6 mothers on TVA-enriched or control diet were nursed by FVB/NJ foster mothers on either diet (Fig. 5A). Serum TVA was detected only in pups with postnatal exposure (TVA-to-TVA and CON-to-TVA) groups, but not in control pups (CON-to-CON), nor in pups with prenatal exposure alone (TVA-to-CON) (fig. S15A). Compared to controls, pups exposed to TVA postnatally (TVA-to-TVA and CON-to-TVA) showed enhanced survival (Fig. 5B) with reduced clinical score in response to influenza viral exposure (fig. S15B–C). These pups also exhibited reduced viral load in lungs (fig. S15D) with increased population and activation states of lung-infiltrating CD4^+^ and CD8^+^ T cells, whereas other immune cell populations were unchanged (Fig. 5C, S15E–H). In contrast, prenatal TVA exposure alone (TVA-to-CON) had no effects. These results together showed that postnatal exposure of TVA *via* breastfeeding was the predominant driver of neonatal T cell reprogramming.

Early-life immune imprinting describes how exposures to environmental cues such as dietary factors during infancy and early childhood impact immune development and have long-lasting effects on immune responsiveness and disease susceptibility(26). Neonatal CD4^+^ naïve T cells contribute to the foundation of adaptive immunity and play a crucial role in early-life immune imprinting shaping lifelong immunity(27). To examine whether postnatal TVA exposure through breastfeeding had an immune imprinting effect, we fed control diet to both TVA-pups and controls after weaning at three weeks (TVA-CON and CON-CON, respectively) and performed the mouse model of lung infection with influenza A virus treatment when the mice reached adulthood at 6–10 weeks (Fig. 5D, *upper*). TVA-pups (3 weeks) accumulated serum TVA, which became undetectable at adulthood after being fed on control diet post-weaning (Fig. 5E). However, following influenza A infection, mice in the group of TVA-CON showed enhanced survival (Fig. 5F) with reduced clinical score upon viral infection (fig. S16A–B). We examined the adult naïve CD4^+^ T cells from mice in both groups (Fig. 5D, *lower*) and found increased CTCF activity (Fig. 5G, fig. S16C) and enhanced Th1 polarization (Fig. 5H) in cells from mice in the TVA-CON group, compared to CON-CON mice. Similarly, TVA exposure *via* breastmilk during postnatal weeks 0–3 improved clinical scores and survival of 8–12-week-old adult mice following influenza A virus challenge (fig. S16D–F).

To determine whether TVA exposure must occur within a defined developmental window to imprint adult immunity, we shifted TVA exposure to weeks 5–8 after birth, and assessed antiviral responses at 10–12 weeks (Fig. 5I). TVA feeding during weeks 5–8 led to detectable serum levels at 8 weeks, which returned to baseline by weeks 10–12 after switching to the control diet at 8 weeks (Fig. 5J). However, this TVA exposure did not enhance resistance to influenza A virus infection (Fig. 5K, S16G–H). Naïve CD4^+^ T cells isolated from these adult mice failed to exhibit the Th1 polarization advantage observed following neonatal TVA exposure (Fig. 5L). *Ex vivo* analyses showed that TVA increased Th1 polarization in neonatal, but not adult naïve CD4^+^ T cells (Fig. S16I–J). We concluded that TVA-mediated immune imprinting was restricted to a critical neonatal window, and that exposure during adulthood was insufficient to reprogram naïve CD4^+^ T cell fate or confer long-term antiviral protection (fig S17).

## Discussion

Our findings identify maternal trans-vaccenic acid as a systemic signal linking maternal diet to neonatal immune development in mice. Although breast milk is widely viewed as a complex system whose benefits arise from the interaction of many components, our study shows that a single dietary molecule can be sufficient to program neonatal T cell development, immunity, and long-term immune imprinting. These results reveal a direct molecular pathway through which maternal nutrition shapes infant immune trajectories. However, our current human dataset does not clarify whether the association between milk TVA and immune outcomes differs between term and preterm infants. Future studies incorporating term infants and explicitly testing gestational-age–dependent effects are therefore warranted.

Compared to adult T cells, neonatal T cells show slower responses to influenza infection with defective migration into infected lungs(28). We found that TVA promoted a shift to neonatal Th1 polarization and reduced neonatal mortality rate upon viral infection. Given that immune polarization in human neonates is influenced by multiple factors, including tissue environment, developmental stage, and postnatal exposures, rather than merely reflecting the uniform Th2 bias observed in mouse neonates, our work addresses this gap by integrating mechanistic insights from murine studies with analyses of human preterm infant samples, offering translational insight into how maternal nutrition shapes early immune trajectories. We also identified cell type-specific functional and regulatory differences between neonatal naïve and effector T cells. Future studies are warranted to define how GPR43 selectively signals through CTCF in neonatal naïve CD4^+^ T cells.

Our study suggests breastfeeding as a critical developmental window during which neonatal immune cells can be reprogrammed to yield durable imprinting effects that shape immunity in adulthood. We propose that TVA acts selectively on neonatal naïve CD4^+^ T cells, the foundation of adaptive immunity, thereby providing a mechanistic link between maternal milk nutrients and early-life immune programming. Notably, the benefit is temporally restricted to the breastfeeding period in mice (0–3 weeks of age): TVA exposure confined to this window enhances adult immune responses, whereas delayed exposure failed to elicit comparable effects. These observations suggest that TVA-dependent imprinting is gated by a narrow developmental timeframe. Whether earlier withdrawal of TVA, or discrete sub-windows within 0–3 weeks, is sufficient to establish durable programming remains unresolved and will require future investigation.

Our findings hint that a diet including foods with high TVA levels, or dietary supplementation of TVA, might be beneficial during pregnancy and lactation. This possibility should be interpreted cautiously, given the well-established adverse health effects of industrial trans fats. However, industrial trans fats such as elaidic acid differ from naturally occurring ruminant trans fats such as TVA in source and composition and may therefore have distinct biological effects. In mice, an elaidic acid–rich diet promotes atherosclerotic lesion development, whereas a TVA-rich butter diet doesn’t increase plaque burden and is less atherogenic under hypercholesterolemic conditions(29). Consistent with this distinction, prospective meta-analyses generally report no association between typical ruminant trans-fat intake and coronary heart disease risk(30). Epidemiological studies further suggest that circulating TVA is associated with lower adiposity, reduced diabetes risk, and lower systemic inflammation, and in rodent models of dyslipidemia, a TVA-enriched diet lowers circulating triglycerides(*31–33*). Furthermore, it is possible that TVA-supplemented infant formula, an ingredient not currently included, may enhance immune system maturation, strengthen early-life immune responses, and promote durable immune imprinting.

## Materials and Methods

### Primary cells

Mouse neonatal or adult naïve T cells were isolated from the spleen and peripheral lymph nodes (PLN) of 10-14 days old or 8-12 weeks old C57BL/6 mice by magnetic bead purification using Easy Sep^™^ Mouse Naïve T Cell Isolation Kit according to the manufacturer’s instructions (Stem Cell Technologies). Cells were cultured in Click’s media at 37° and 5% CO_2_ incubator for further experiments.

### Mice

Animal experiments were conducted and designed according to protocols approved by the Institutional Animal Care and Use Committee of The University of Chicago. Mice were housed and bred at the University of Chicago Animal Resource Center in specific pathogen-free conditions. Mice were on 12-hour light/dark cycles that coincided with daylight in Chicago, IL, USA. The housing facility was maintained at 20-25°C and 30-70% humidity. Mice were euthanized by carbon dioxide inhalation followed by a secondary physical method to ensure death. C57BL/6J (The Jackson Laboratory, JAX:000664; RRID: IMSR_JAX:000664), TCRα Knock-out (B6.129S2-Tcratm1Mom/J) (The Jackson Laboratory, JAX:002116; RRID: IMSR_JAX:002116), FVB/NJ (The Jackson Laboratory, JAX: 001800; RRID: IMSR_JAX:001800), *Tbx21*^−/−^ (B6.129S6-Tbx21^tm1Glm^/J) (The Jackson Laboratory, JAX: 004648; RRID: IMSR_JAX:004648), CD4 KO (B6.129S2-Cd4^tm1Mak^/J) (The Jackson Laboratory, JAX: 002663; RRID: IMSR_JAX:002663), CD4Cre (B6.Cg-Tg(Cd4-cre)1Cwi/BfluJ) (The Jackson Laboratory, JAX: 022071; RRID: IMSR_JAX:022071), B6 CD45.1 (B6.SJL-Ptprc^a^ Pepc^b^/BoyJ) (The Jackson Laboratory, JAX: 002014; RRID: IMSR_JAX:002014) mice were purchased from The Jackson Laboratory. *Gpr43^fl/fl^* and *Gpr43^−/−^* mice were kindly provided by Dr. Brian Layden. Sex-and age-matched mice were used throughout the study, and both male and female mice were used. The genetically modified mice were viable and developed normally. Mice were randomly assigned to experimental groups with relevant variables balanced when applicable. Investigators were blinded to group allocation during outcome assessment and data analysis whenever feasible. Inclusion and exclusion criteria were predefined, and animals were excluded only for technical or health-related reasons unrelated to the experimental outcome. No other data points were excluded.

### Human samples

Blood spot cards of preterm infants and paired milk samples from their moms or donors were provided by Dr. Erika C. Claud from the Microbiome In Neonatal Development (MIND) study. For the preterm infants, breastmilk intake did not differ between infants with and without PBD, gestational age was comparable between these groups, and the amount of TVA in donor milk was similar to that in mothers’ own breastmilk. The MIND study recruits preterm infants (infants born < 37 weeks postmenstrual age) from the University of Chicago Comer Children’s Hospital after retrieving written informed consent from a parent. Exclusion criteria included the presence of genetic syndromes, severe congenital anomalies, or nonviability. All sample collection and study procedures were approved by the University of Chicago Institutional Review Board (IRB16-1431), which abides by the Declaration of Helsinki. Detailed sample information in Data S10.

### TVA measurement of infant blood spot card samples

The blood spot cards of the preterm infants were used for TVA level detection. 3.2-mm (1/8-inch) diameter disks (11-24 disks) were punched from dried infant blood-spots in a 20 mL glass vial and spike 10 μL of ^13^C_5_-trans vaccenic acid and extracted with the modified Matyash et al method(34). In brief, the disks were extracted with 800 μL of ice-cold methanol (containing 0.1 mg/mL butylated hydroxytoluene-BHT) and sonicated for 15 minutes, followed by the addition of 2400 μL of methyl-tert-butyl ether and sonicated for 45 minutes at room temperature and centrifuged at 18,000g for 15 minutes. The upper 2 mL of the organic layer was dried using Genevac 2.4 EZ-elite evaporator and stored at −20°C until analysis. On the day of the LC-MS analysis, samples were re-suspended in 80 μL of 3/2/1 isopropanol/acetonitrile/water, sonicated for 2 minutes, thermomixer for 10 minutes at 15 °C. The supernatant was transferred to the LC-MS vial after spinning it down at 18,000g for 20 minutes at 15°C. Chromatography separation was performed using the Cortecs T3 (2.1x100mm, 1.6micron, part # 186008499 Waters Corporation) column connected to a Vanquish Horizon UHPLC system and IQ-X tribrid mass spectrometers. The column temperature, injection volume, and flow rate were 40°C, 5 μL, and 0.3 mL/minute, respectively. The mobile phase A (MPA) was 60/40 acetonitrile/water, 10 mM ammonium formate+0.1% formic acid, and MPB was 89.1/9.9/0.99 isopropanol/acetonitrile/water, 10 mM ammonium formate +0.1% formic acid. The chromatographic gradient was 0 minute: 5%B, 1.2 minute: 5%B, 5 minutes: 50%B, 9.00 minute: 50%B, 13.00 minute: 70%B, 15.5 minute: 70%B, 17.00 minute: 100%B, 20.00 minute: 100%B, 20.2 minute: 5%B, 28.00 minute: 5%B. MS1 parameters were as follows: spray voltage: 2800 V negative, sheath gas: 40, auxiliary gas: 10, sweep gas: 1, ion transfer tube temperature: 300°C, vaporizer temperature: 350°C, orbitrap resolution: 60K, scan range(m/z): 120-500, RF lens (%): 60, automatic gain control (AGC) target: 100%, and a maxIT of 118 milliseconds (ms). The TVA levels were normalized to an added stable isotope standard-^13^C_5_ trans vaccenic acid, and calculated for the 10 disks.

### The mouse model to test the effects of TVA on the neonatal immune system

Establishing TVA-feeding mouse lines: For each mouse line, we start to feed 8-week-old C57BL/6J males and females with the TVA-enriched diet or control diet for two weeks before pairing them for breeding. Their offspring were kept on the TVA-enriched diet or control diet and inbred to continue this line. After the control diet and TVA diet mouse lines stabilized (2-3 generations), the breeders and neonatal pups of each group were used for further analysis. To avoid potential transgenerational effects, we also had acute TVA exposure paradigms that restricted parental dietary exposure to the first generation. Specifically, we used 8-week-old male and female C57BL/6J mice and established breeders under two new dietary timing regimens:1. Diet switch at breeder setup (TVA or control diet initiated on the day breeders were paired); 2. Late-gestation switch (diet changed 3 days prior to delivery, E17). In these experiments, pups remained with their biological mothers in both the CON and TVA diet groups. For each condition, we used more than five breeder cages, and pups were collected from multiple independent cages. To ensure robustness and minimize cage-specific effects, we performed three to five independent experiments, each including five or more mice per group. These experimental designs reduce potential cage effects and ensure the reliability of the statistical analyses.

For mouse milk collection, breeders were separated from their 1- or 2-week-old pups for 1 hour. Following this, each breeder received a 1 IU intraperitoneal (IP) injection of oxytocin. Fifteen minutes later, milk samples were collected from the breeders. Blood samples were also collected from pups aged 1 to 5 weeks from each group. Additionally, BW, spleen weight, and thymus weight were monitored. Spleen, thymus, and lung tissues were further analyzed for immune cell profiling. Spleen samples from 2-week-old pups in each group were also used for single-nuclei multi-omics analysis, including snRNA-seq and snATAC-seq (samples from 5 mice/group pooled).

### Diet formula

Diets for mouse experiments were obtained from Research Diet Inc. Control diet (D12450J), 0.1% TVA diet (D23031402), 1% TVA diet (D19110701).

### Mouse splenocytes isolation

Mouse spleens were disrupted with a syringe plunger in 1 mL PBS in a 40 μm strainer, and cells were filtered to a 15 mL tube, then the cells were washed with PBS, and centrifuged at 300×g for 5 minutes. The resulting cell pellets were re-suspended with 2 mL red cell lysis buffer (Invitrogen), then the cells were incubated at room temperature for 10 minutes and centrifuged at 300×g for 5 minutes after adding 13 mL PBS. The resulting cell pellets were designated splenocytes and used for the following experiments.

### Mouse peripheral lymph node cell isolation

Peripheral lymph nodes were disrupted with a syringe plunger in 1 mL PBS in a 40 μm strainer, and cells were filtered to a 15 mL tube, then the cells were washed with PBS, and centrifuged at 300×g for 5 minutes. The resulting cell pellets were designated lymph node cells and used for the following experiments.

### Mouse thymus single-cell isolation

Mouse thymuses were disrupted with a syringe plunger in 1 mL PBS in a 40 μm strainer, and cells were filtered to a 15 mL tube, then the cells were washed with PBS, and centrifuged at 300×g for 5 minutes. The resulting cell pellets were re-suspended with 2 mL red cell lysis buffer (Invitrogen), incubated at room temperature for 10 minutes, and centrifuged at 300×g for 5 minutes after adding 13 mL PBS. The resulting cell pellets were designated thymus cells and used for the following experiments.

### Primary naïve CD8^+^ or naïve CD4^+^ T cell isolation and activation

Mouse primary naïve CD8^+^ or CD4^+^ T cells were isolated from the spleen and peripheral lymph nodes (PLN) of C57BL/6 mice by magnetic bead purification using EasySep^™^ Mouse naïve CD8^+^ or CD4^+^ T Cell Isolation Kit according to the manufacturer’s instructions (Stemcell Technologies). Isolated primary CD8^+^ or CD4^+^ T cells were activated *in vitro* for 18 hours with plate-bound anti-CD3 (1 μg/mL; Biolegend) and anti-CD28 (0.5 μg/mL; Biolegend) antibodies in Click’s media at 37° and 5% CO_2_ incubator. Activated CD8^+^ or CD4^+^ T cells were ready for further experiments. A naïve T cell control was maintained in Click’s media containing 10 ng/mL IL-7 (BioLegend).

### Milk IgA, lactoferrin, IL-7, and IL-1β levels

After collecting milk samples from each group (five samples per group), 10 μL of each sample was used for IgA level detection by Mouse IgA ELISA Kit (Invitrogen, 88-50450-88), 10 μL of each sample was used for lactoferrin level detection by Mouse LTF/Lactoferrin ELISA Kit (Invitrogen, EEL103), 10 μL of each sample was used for IL-7 level detection by Mouse IL-7 ELISA Kit (Invitrogen, EMIL7), and 10 μL of each sample was used for IL-1β level detection by Mouse IL-1 beta ELISA Kit (Invitrogen, BMS6002), according to the manufacturer’s instructions.

### *Ex-vivo* assay to assess the function of naïve CD4^+^ T cells from neonatal mice

TVA diet-fed and CON diet-fed C57BL/6J and *Gpr43*^−/−^ mouse lines were generated as described above. Neonatal offspring were harvested at postnatal day 10, and naïve CD4^+^ T cells were isolated for the following assays: Th polarization: Cells were seeded on plates precoated with anti-CD3 (1 μg/mL) and anti-CD28 (0.5 μg/mL) and incubated for 12 hours. Thereafter, Th1, Th2, or Th17 differentiation cocktails were added, and cells were cultured for 48 hours. The frequency of polarized Th subsets was determined by flow cytometry: T-bet^+^IFNγ^+^CD4^+^ as Th1, GATA3^+^IL-4^+^CD4^+^ as Th2, RORγ^+^IL17^+^CD4^+^ as Th17. Early activation (CD69) assay: Naïve CD4^+^ T cells were stimulated on anti-CD3/CD28-coated plates (same coating concentrations) for 6 hours, then stained for CD69 and analyzed by flow cytometry. Proliferation assay: Following a 12-hour activation on anti-CD3/CD28-coated plates, cells were transferred to fresh medium containing 5 IU/mL human IL-2, 1 ng/mL mouse IL-7, and 5 ng/mL mouse IL-15, and cultured for an additional 48 hours. Cell expansion was assessed by counting or proliferation dye dilution.

### *In-vitro* treatment for naïve CD4^+^ T cells

Naïve CD4^+^ T cells were isolated from neonatal (10–14 days old) or adult (8–12 weeks old) C57BL/6J mice. Cells were treated with 10 ng/mL mouse IL-7 in the presence of either DMSO, 20 μM TVA, oleic acid, linoleic acid, palmitoleic acid, elaidic acid, or conjugated linoleic acid (cis-9, trans-11-octadecadienoic acid) for 24 hours. Following treatment, cells were stimulated on anti-CD3/CD28-coated plates for 12 hours. Subsequently, Th1 differentiation cocktails were added, and cells were cultured for an additional 48 hours. The frequency of Th1-polarized cells was then assessed by flow cytometry.

Similarly, naïve CD4^+^ T cells were isolated from neonatal (10–14 days old) *Gpr43*^−/−^ mice and treated with 10 ng/mL mouse IL-7 along with DMSO or 20 μM TVA for 24 hours, followed by anti-CD3/CD28 stimulation for 12 hours. Th1 differentiation cocktails were then applied, and cells were cultured for 48 hours. Th1 polarization was analyzed by flow cytometry.

To examine the role of cAMP-PKA signaling, naïve CD4^+^ T cells from neonatal (10–14 days old) C57BL/6J mice were treated with 10 ng/mL IL-7 and either DMSO, 20 μM TVA, 200 nM PKA inhibitor H-89 dihydrochloride, or 1 μM 8-Bromo-cAMP for 24 hours. Cells were then stimulated with anti-CD3/CD28 for 12 hours, followed by Th1 polarization for 48 hours. Th1 cell frequency was assessed by flow cytometry.

To investigate transcriptional regulation, naïve CD4^+^ T cells from neonatal (10–14 days old) C57BL/6J mice were treated with Accell si*Ctcf* (Horizon, E-044693-00-0020), Accell si*Creb1* (Horizon, E-040959-00-0020), or Accell control siRNA (Horizon, D-001910-10-05) according to the manufacturer’s protocol. After confirming knockdown efficiency, cells were treated with DMSO or 20 μM TVA for 24 hours, followed by 12-hour anti-CD3/CD28 stimulation and 48-hour Th1 differentiation. The frequency of Th1 cells was analyzed by flow cytometry.

### Electrophoretic Mobility Shift Assay (EMSA) for CTCF Activity

CTCF DNA-binding activity was assessed using the Pierce^™^ LightShift^™^ Chemiluminescent EMSA Kit (Thermo Fisher Scientific, cat# 20148) according to the manufacturer’s instructions. A biotin-labeled double-stranded DNA oligonucleotide probe containing a known CTCF-binding motif was synthesized (Integrated DNA Technologies) with the following sequence:5′-gtagtgacccCGgagccaCGtggagccaccttcagacccagagctgaagcaggagggtgacCGg-3′ (biotin-dT at the 5′ end of the reverse strand). For *in vitro* treatment assays, naïve CD4^+^ T cells were isolated from five 10-day-old C57BL/6J mice per sample. Cells were treated with 10 ng/mL recombinant mouse IL-7 in the presence of DMSO (vehicle), 20 μM TVA, 200 nM PKA inhibitor H-89 dihydrochloride, or 1 μM 8-Bromo-cAMP for 24 hours. Nuclear proteins were then extracted using the NE-PER^™^ Nuclear and Cytoplasmic Extraction Reagents (Thermo Fisher Scientific, cat# 78833). For imprinting experiments in adult mice, naïve CD4^+^ T cells were isolated from TVA-to-CON and CON-to-CON adult mice (five mice per group). Nuclear extracts were prepared as described above. Equal amounts of nuclear protein (quantified by BCA assay) were incubated with the biotin-labeled DNA probe and subjected to non-denaturing polyacrylamide gel electrophoresis. DNA-protein complexes were transferred to a nylon membrane, detected by chemiluminescence, and quantified by densitometry to evaluate the ratio of CTCF-DNA complex/ free probe to indicate CTCF activity.

### Neonatal mouse model for influenza A virus challenge

C57BL/6J neonatal mice from the control or TVA groups were challenged with influenza A virus (H1N1; A/Puerto Rico/8/1934, PR8). Ten-day-old pups from either the TVA or control diet groups were infected intranasally with PR8 at 0.04 TCID50/g BW in 7 μL sterile saline. Pups were weighed and monitored daily for activity, respiratory rate, and maternal interaction. Neonates that were ignored by the dam, exhibited weight loss, or showed minimal spontaneous movement were removed from the cage per humane monitoring criteria. For survival studies, pups were followed for 14 days post-infection, and clinical scores were recorded daily using the following scale: 0, no signs of disease (active, well groomed, feeding normally; normal breathing); 1, mild disease (slightly ruffled fur; slight reduction in activity; subtle weight loss, <5% of expected BW); 2, moderate disease (noticeable ruffled fur; reduced activity and responsiveness; mild dehydration; 5–10% weight loss); 3, severe disease (marked ruffling and hunched posture; substantial reduction in movement/responsiveness; labored breathing including tachypnea or dyspnea; 10–20% weight loss); 4, moribund (unresponsive to stimuli; cyanosis and/or visibly labored breathing; severe dehydration; >20% weight loss); 5, death.

For endpoint analyses, cohorts were euthanized on days 4, 7, or 10 post-infection to assess viral burden, lung pathology, and immune responses by flow cytometry. Flow panels included leukocyte lineage markers (CD45, CD3, CD4, CD8, TCRδ, NK1.1, CD11b, Ly6G, F4/80, Ly6C, MHC II, CD11c, B220, Foxp3) and T cell activation/virus-reactive markers (CD49d, CD11a, CD44, CD69) as well as influenza-specific CD8^+^ T cell tetramers (NP_331–339_ and PA_224–233_). For intracellular cytokine staining, lung single-cell suspensions were stimulated *ex vivo* with PMA in the presence of brefeldin A for 6 h, followed by detection of IFN-γ, T-bet, and TNF-α in CD4^+^ T cells and IFN-γ and TNF-α in CD8^+^ T cells. In addition, lungs were collected on day 7 post-infection for single-nucleus multi-omics (snRNA-seq and snATAC-seq), with samples from five mice per group pooled for each analysis.

### Mouse lung infiltrating cell isolation

Neonatal C57BL/6 mice challenged with Influenza A virus were euthanized, placed in a supine position, and secured with pins. The chest area was disinfected with 70% ethanol. Using sterile scissors, the thoracic cavity was carefully opened to expose the heart. A 23-G needle was inserted into the right ventricle, and 10–15 mL of ice-cold PBS was slowly perfused to flush out circulating blood. Lungs were carefully dissected, rinsed in PBS to remove residual blood, and transferred to a Petri dish containing 5 mL of RPMI-1640 supplemented with 2% fetal bovine serum (FBS). Lung tissues were minced into small fragments using sterile scissors and transferred to a 15 mL conical tube containing 5 mL of digestion buffer (RPMI-1640 supplemented with 1 mg/mL Collagenase D and 100 U/mL DNase I). The tissue suspension was incubated at 37°C for 30–40 minutes with gentle shaking every 5–10 minutes. Following digestion, the cell suspension was passed through a 70 μm cell strainer into a 50 mL conical tube to obtain a single-cell suspension. The strainer was subsequently washed with an additional 5 mL of RPMI-1640 containing 2% FBS. Red blood cells were lysed by incubating the suspension with RBC lysis buffer for 2–3 minutes at room temperature. The cells were then washed with RPMI-1640 and centrifuged at 300 × g for 5 minutes at 4 °C. The resulting cell pellet was designated as lung-infiltrating cells and used for subsequent experiments.

### Mouse lung IL-1β and TNF-α levels

Lung tissues were collected from mice at 4, 7, and 10 days post influenza A virus challenge, and rinsed briefly in cold PBS to remove blood contaminants. Tissues were weighed and homogenized in ice-cold PBS supplemented with a protease inhibitor cocktail (Roche, Complete Mini) using a bead mill homogenizer. Homogenates were centrifuged at 12,000 × g for 15 minutes at 4°C, and the supernatants were collected for analysis. IL-1β and TNF-α levels in the lung homogenates were measured using a commercially available mouse IL-1β ELISA kit (Biolegend, 432601) and TNF-α ELISA kit (Biolegend, 430901) according to the manufacturer’s instructions.

### Mouse serum TNF-α level

Blood samples were collected from mice at 4, 7, and 10 days post influenza A virus challenge. Samples were centrifuged at 2,000 × g for 10 minutes at 4°C to isolate the serum. The supernatant (serum) was carefully collected and stored at −80°C until analysis. Serum TNF-α levels were quantified using a commercially available mouse TNF-α ELISA kit (Biolegend, 430901) according to the manufacturer’s instructions.

### Single nuclei Multiome for spleen

Single nuclei Multiome library preparation, sequencing, and analysis were conducted by Singulomics Corporation (https://singulomics.com/, Bronx, NY). Single nuclei gene expression libraries and single nuclei ATAC libraries were constructed from nuclei isolated from cryopreserved mouse spleen cells on the 10x Genomics Chromium System using the Chromium Next GEM Single Cell Multiome ATAC + Gene Expression Reagent Bundle. Cryopreserved, viable single cell suspensions were thawed, washed, resuspended in cell culture media with 0.04% BSA, and counted. The cells were lysed and permeabilized with Nonidet P40 Substitute, Digitonin, and Tween-20. The isolated nuclei were purified, centrifuged, and resuspended in Diluted Nuclei Buffer. The nuclei were then loaded into the Chromium Controller (10x Genomics, Pleasanton, CA) to encapsulate single nuclei into droplet emulsions following the manufacturer’s recommendations. 6,000 nuclei were targeted to be captured per sample. Library preparation was performed according to the Chromium Next GEM Single Cell Multiome ATAC + Gene Expression User Guide. Amplified cDNAs and the libraries were measured by Qubit dsDNA HS assay (Thermo Fisher Scientific, Wilmington, DE) and quality assessed by TapeStation (Agilent Technologies, Santa Clara, CA). Libraries were sequenced on a NovaSeq 6000 instrument (Illumina, San Diego, CA, United States), and reads were subsequently processed using 10x Genomics Cell Ranger ARC (v 2.0.2) analytical pipeline and mouse mm10 (2020-A) reference. Dataset aggregation was performed using the cellranger-arc aggr function, normalizing for the total number of confidently mapped reads across libraries.

### Single nuclei Multiome for lung

Single nuclei Multiome library preparation, sequencing, and analysis were conducted by Singulomics Corporation (https://singulomics.com/, Bronx, NY). Single nuclei gene expression libraries and single nuclei ATAC libraries were constructed from nuclei isolated from flash-frozen mouse lung tissue samples on the 10x Genomics Chromium System using the Chromium Next GEM Single Cell Multiome ATAC + Gene Expression Reagent Bundle. Flash-frozen mouse lung tissue samples were homogenized, lysed, and permeabilized with Nonidet P40 Substitute, Digitonin, and Tween-20. The isolated nuclei were purified, centrifuged, and resuspended in Diluted Nuclei Buffer. The nuclei were then loaded into the Chromium Controller (10x Genomics, Pleasanton, CA) to encapsulate single nuclei into droplet emulsions following the manufacturer’s recommendations. 7,000 nuclei were targeted to be captured per sample. Library preparation was performed according to the Chromium Next GEM Single Cell Multiome ATAC + Gene Expression User Guide. Amplified cDNAs and the libraries were measured by Qubit dsDNA HS assay (Thermo Fisher Scientific, Wilmington, DE) and quality assessed by TapeStation (Agilent Technologies, Santa Clara, CA). Libraries were sequenced on a NovaSeq 6000 instrument (Illumina, San Diego, CA, United States), and reads were subsequently processed using 10x Genomics Cell Ranger ARC (v 2.0.2) analytical pipeline and mouse mm10 (2020-A) reference. Dataset aggregation was performed using the cell ranger-arc aggr function, normalizing for the total number of confidently mapped reads across libraries.

### Single nuclei Multiome data analysis

All data were generated using the 10x Chromium Single Cell Multiome platform. For each sample, the analysis input is the Cell Ranger ARC output, then we applied Seurat v5.1.0(35) and Signac v 1.13.0(36) to create a multi-omics Seurat object with paired gene expression and DNA accessibility profiles for each sample. The 10x multiome workflow encompasses quality control (QC), dimensionality reduction, clustering, and UMAP visualization across RNA, ATAC, and a joint neighbor graph. This graph represents both gene expression and DNA accessibility measurements via weighted nearest neighbor (WNN) methods(37). The workflow also includes cell type annotations, gene activity assessment, peak calling using MACS2(38), and the identification and visualization of condition-specific candidate cis-regulatory elements (cCREs), differentially active motifs, and key regulators. For each sample, we conducted QC, dimensionality reduction, clustering, and cell type annotations, followed by the integration of all samples for downstream analysis. For each gene and cell, promoter accessibility was quantified as the sum of Tn5-corrected ATAC fragments overlapping the promoter region, defined as transcription start site (TSS) ±2 kb (GENCODE vM25, mm10). Fragments were deduplicated, offset by +4/−5 bp to adjust for transposase integration, and filtered to exclude mitochondrial and ENCODE blacklist regions. Promoter sums were normalized either as counts per million (CPM) or by term frequency–inverse document frequency (TF-IDF) prior to downstream analyses(*36, 39*). Cluster-level values represent the mean promoter sum across cells within each cluster. Then, TF “activity” was predicted based on the presence of binding motifs within DA chromatin regions using chromVAR(40), and the positional weight matrix (PWM, refer to https://genome.ucsc.edu/goldenPath/help/hgRegMotifHelp.html) was obtained from the JASPAR2020 database(41). Paired data provides an opportunity to identify transcription factors (TFs) that satisfy multiple criteria, helping to narrow down the list of putative regulators to the most likely candidates. We aim to identify TFs whose expression is enriched in multiple cell types in the RNA measurements and with enriched accessibility for their motifs in the ATAC measurements. To do this, we use the “presto” package (detailed in https://immunogenomics.github.io/presto/articles/getting-started.html) to perform fast differential expression in gene expression data and chromVAR motif accessibilities. presto calculates an “AUC” statistic, which reflects the power of each gene (or motif) to serve as a marker of cell type. A maximum AUC value of 1 indicates a perfect marker. Since the AUC statistic is on the same scale for both genes and motifs, we calculate the average of the AUC values from the two tests and use this to rank TFs for each cell type. Then, we visualize the TF expression (RNA level), gene activity (ATAC level), and motif activity (chromVAR) of the key regulators.

### TCRα KO mouse model

8-week-old TCRα KO male and female mice were fed with the TVA-enriched diet or control diet for two weeks before pairing them for breeding. Their offspring were kept on a TVA-enriched diet or a control diet and inbred to continue this line. After the control diet and TVA diet mouse lines stabilized (2-3 generations), the neonatal pups of each group were used for further analysis. 10-day-old neonatal mice from either the TVA or control diet groups were intranasally infected with Influenza virus PR8 (0.01 TCID50/g in 7 μL saline). The pups were weighed and monitored daily for activity levels, respiratory rate, and maternal interaction. Neonates that were ignored by the mother, exhibited weight loss, or showed a lack of movement were removed from the cage. The neonates were observed for two weeks for survival experiments.

### CD4 KO mouse model

8-week-old CD4 KO male and female mice were fed with the TVA-enriched diet or control diet for two weeks before pairing them for breeding. The neonatal pups of each group were used for further analysis. 10-day-old neonatal mice from either the TVA or control diet groups were intranasally infected with Influenza virus PR8 (0.01 TCID50/g in 7 μL saline). The pups were weighed and monitored daily for activity levels, respiratory rate, and maternal interaction. Neonates that were ignored by the mother, exhibited weight loss, or showed a lack of movement were removed from the cage. The neonates were observed for two weeks for survival experiments.

### *Gpr43*^−/−^ mouse model

8-week-old *Gpr43*^−/−^ male and female mice were fed with the TVA-enriched diet or control diet for two weeks before pairing them for breeding. Their offspring were kept on a TVA-enriched diet or a control diet and inbred to continue this line. After the control diet and TVA diet mouse lines stabilized (2-3 generations), the neonatal pups of each group were used for further analysis. 10-day-old neonatal mice from either the TVA or control diet groups were intranasally infected with Influenza virus PR8 (0.04 TCID50/g in 7 μL saline). The pups were weighed and monitored daily for activity levels, respiratory rate, and maternal interaction. Neonates that were ignored by the mother, exhibited weight loss, or showed a lack of movement were removed from the cage. The neonates were observed for two weeks for survival experiments.

### *Gpr43*^fl/fl^CD4^Cre^ mouse model

8-15 weeks old *Gpr43*^fl/fl^ mice were crossed with CD4^cre^ mice to generate *Gpr43*^fl/fl^CD4^Cre^ and littermate control mice. Breeders were fed with the TVA or CON diet. The neonatal pups of each group were used for further analysis. 10-day-old neonatal mice from each group were intranasally infected with Influenza virus PR8 (0.04 TCID50/g in 7 μL saline). The pups were weighed and monitored daily for activity levels, respiratory rate, and maternal interaction. Neonates that were ignored by the mother, exhibited weight loss, or showed a lack of movement were removed from the cage. The neonates were observed for two weeks for survival experiments.

### *T-bet^−/−^ (Tbx21^−/−^)* mouse model

8-week-old *Tbx21^−/−^* male and female mice were fed with the TVA-enriched diet or control diet for two weeks before being paired for breeding. Their offspring were kept on a TVA-enriched diet or a control diet and inbred to continue this line. After the control diet and TVA diet mouse lines stabilized (2-3 generations), the neonatal pups of each group were used for further analysis. 10-day-old neonatal mice from either the TVA or control diet groups were intranasally infected with Influenza virus PR8 (0.02 TCID50/g in 7 μL saline). The pups were weighed and monitored daily for activity levels, respiratory rate, and maternal interaction. Neonates that were ignored by the mother, exhibited weight loss, or showed a lack of movement were removed from the cage. The neonates were observed for two weeks for survival experiments.

### Adoptive transfer mouse model

CD45.1 and CD45.2 C57BL/6 mice were used to generate donor pups distinguished by congenic markers. For the CON donor group, 8–12-week-old CD45.1 male and female mice were established as breeders (5 pairs) and maintained on the control (CON) diet. In parallel, for the TVA donor group, congenic-mixed breeding pairs (5 pairs) were established on the TVA diet by crossing CD45.1 and CD45.2 mice (either CD45.1 males × CD45.2 females or CD45.1 females × CD45.2 males), generating pups carrying CD45.1/2 markers. Donor pups from the CON group (CD45.1, n = 40 total pups) and TVA group (CD45.1/2, n = 38 total pups) were used at postnatal day 10–15. Neonatal naïve CD4^+^ T cells were isolated from each group using a negative selection kit according to the manufacturer’s instructions. Purified neonatal naïve CD4^+^ T cells from CON-breastfed CD45.1 pups and TVA-breastfed CD45.1/2 pups were then mixed at a 1:1 ratio, and the final ratio was confirmed by flow cytometry prior to injection.

To generate recipient pups, 8–12-week-old CD45.2 male and female mice were established as breeders (5 pairs) on the CON diet 2 weeks prior to setting up the donor breeders. Recipient pups from these CD45.2 pairs were used for adoptive transfer. When recipient pups reached 2 weeks of age, endogenous CD4^+^ T cells were depleted by intraperitoneal injection of anti-CD4 (GK1.5; 100 μg per mouse) (Day −14). Two weeks later (recipient age: 4 weeks), the mixed neonatal naïve CD4^+^ T cells (CD45.1^+^ and CD45.1/2^+^, 4 million cells per mouse) were transferred into recipient mice (13 recipients total) via intravenous (tail vein) injection (Day 0). All recipient mice were maintained on the CON diet throughout the experiment.

At 1 day post–adoptive transfer (Day 1), recipient mice were infected intranasally with influenza A virus PR8 at 0.05 TCID50/g BW in 10 μL saline. At 7 days post-infection (Day 8), spleen and lung tissues were harvested for flow cytometric analyses. In the spleen, the relative contribution of donor populations was assessed by quantifying the percentages of CD45.1^+^ versus CD45.1/2^+^ cells within the donor-derived CD4^+^CD45.1^+^ gate. In the lung, donor Th1 differentiation was evaluated by quantifying CD45.1^+^ versus CD45.1/2^+^ cells within CD4^+^T-bet^hi^CD45.1^+^ donor cells. The competitive fitness of TVA-derived donor cells relative to CON-derived donor cells was further summarized as the ratio of CD45.1/2^+^ (TVA) to CD45.1^+^ (CON) donor cells in both spleen and lung of each recipient.

### TVA measurement, and lipid (fatty acid) profile of serum and milk samples

The twenty-two preterm infants (with an Estimated Gestational Age of 24-33 weeks) were divided into two groups based on whether they were diagnosed with Bronchopulmonary Dysplasia (BPD), which was determined by the need for supplemental oxygen at 36 weeks postmenstrual age. The paired milk from the infants’ mothers or donors was used for fatty acid profiling. Mouse milk samples were also collected from the TVA and CON group mice. Approximately 200 μL of milk sample was spiked with 25 μL of 100 μM of ^13^C_5_-trans vaccenic acid and extracted with methods modified from Matyash et al(34). In brief, the milk sample was mixed with the 800 μL ice-cold methanol containing 0.1 mg/mL butylated hydroxytoluene and sonicated for 15 minutes, followed by the addition of 2400 μL of methyl-tert-butyl ether and sonicated for 30 minutes at room temperature. The phase separation was induced by adding 800 μL of water, sonication for 15 minutes, and centrifuging at 18,000g for 15 minutes. The upper 2000 μL of the organic layer was collected in a scintillation vial, dried down using the Genevac vacuum dryer, and stored at −20°C until analyses. On the day of the LC-MS analysis, samples were re-suspended in 80 μL of 3/2/1 isopropanol/acetonitrile/water, sonicated for 2 minutes, thermomixer for 10 minutes at 15 °C. The supernatant was transferred to the LC-MS vial after spinning it down at 18,000g for 20 minutes at 15°C. The fatty acids were separated on a Cortecs T3 (2.1x100mm, 1.6micron, part # 186008499 Waters Corporation) column connected to a Vanquish Horizon UHPLC system and IQ-X tribrid mass spectrometers. The column temperature, injection volume, and flow rate were 40°C, 5 μL, and 0.3mL/minute, respectively. The mobile phase A (MPA) was 60/40 acetonitrile/water, 10 mM ammonium formate+0.1% formic acid, and MPB was 89.1/9.9/0.99 isopropanol/acetonitrile/water, 10 mM ammonium formate +0.1% formic acid. The chromatographic gradient was 0 minute: 5%B, 1.2 minute: 5%B, 5 minutes: 50%B, 9.00 minute: 50%B, 13.00 minute: 70%B, 15.5 minute: 70%B, 17.00 minute: 100%B, 20.00 minute: 100%B, 20.2 minute: 5%B, 28.00 minute: 5%B. MS1 parameters were as follows: spray voltage: 2800 V negative, sheath gas: 40, auxiliary gas: 10, sweep gas: 1, ion transfer tube temperature: 300°C, vaporizer temperature: 350°C, orbitrap resolution: 60K, scan range(m/z): 120-500, RF lens (%): 60, automatic gain control (AGC) target: 100%, and a maxIT of 118 milliseconds (ms). For the serum samples, 25 μL of serum was extracted with 225μL o methanol (with BHT), 750 μL of MTBE, and 225 μL of water. The fatty acids were identified by matching their retention time to the respective commercially available reference standards.

### Mouse-fostering model

8-week-old male and female C57BL/6 and FVB/NJ mice were fed either a control diet or a 0.1% TVA-supplemented diet for three weeks. Following this, the mice were bred within their respective control or TVA groups. In the cross-fostering experiments, littermate controls were used: pups from the same litter were evenly divided and fostered by CON- or TVA-fed dams. Once both C57BL/6 and FVB/NJ mice gave birth on the same day, 1-day-old pups from C57BL/6 mice on the control diet were randomly and equally assigned to either control or TVA-fed FVB/NJ foster mothers. Similarly, 1-day-old pups from C57BL/6 mice on the TVA diet were randomly and equally assigned to either control or TVA-fed FVB/NJ foster mothers. At 10 days old, the four groups of pups were challenged with influenza A virus. We monitored survival and clinical scores for two weeks, and on day 7 post-infection, lung tissue was collected for virus load and immune cell flow cytometry analysis, which includes cell type markers CD45, CD4, CD8, CD3, TCR-delta, NK1.1, CD11b, Ly6G, F4/80, Ly6C, MHCII, CD11c, B220; CD4 and CD8 activation or influenza virus-specific react markers CD49d, CD11a, CD44, CD69, Tetramer NP_331-325_, Tetramer PA_224-233_.

### Mouse model of immune imprinting effects

Lactational TVA exposure (TVA-milk imprinting): C57BL/6J breeder pairs were maintained on either a TVA diet or a CON diet. Offspring were nursed by their biological dams until weaning at 3 weeks of age, and thus received breastmilk derived from TVA- or CON-fed dams during the lactation period. After weaning, all offspring were switched to the CON diet and maintained until adulthood (6-10 or 8–12 weeks of age). Peripheral blood was collected at weaning (3 weeks) and again in adulthood to quantify circulating TVA levels. Naïve CD4^+^ T cells were isolated from each group for downstream analyses of CTCF transcriptional activity and Th1 polarization capacity. In a separate experiment, adult offspring (6-10 or 8–12 weeks old) were intranasally challenged with influenza A virus PR8 at 0.4 TCID_50_/g BW in 20 μL sterile saline, and mice were monitored daily for survival.

Post-weaning dietary TVA exposure: In an independent cohort, C57BL/6J breeder pairs were maintained on the CON diet. Offspring were nursed until weaning at 3 weeks of age, and all pups were maintained on the CON diet until 5 weeks of age. At 5 weeks, pups were randomly assigned in equal numbers to receive either the TVA diet or the CON diet until 8 weeks of age. At 8 weeks, both groups were switched to the CON diet and maintained until adulthood (10–12 weeks of age). Peripheral blood was collected at 8 weeks and again at 10–12 weeks to quantify circulating TVA levels. Naïve CD4^+^ T cells were isolated for assessment of Th1 polarization capacity. In a separate experiment, adult offspring (10–12 weeks old) were intranasally challenged with influenza A virus PR8 at 0.4 TCID_50_/g BW in 20 μL sterile saline, and mice were monitored daily for survival.

### Mouse serum cytokine array

2-week-old mouse serum samples from each group were used for the cytokine profiling by MILLIPLEX^®^ Mouse Cytokine/Chemokine Magnetic Bead Panel - Immunology Multiplex Assay Kit (Millipore Sigma, MCYTOMAG-70K) according to the manufacturer’s instructions.

### Blood spot card cytokine detection

Six 3.2-mm (1/8-inch) diameter disks were punched from dried blood spots. The disks were eluted together in 180 μL of PBS containing a “Complete protease inhibitor cocktail without EDTA” (Pierce), including 0.5 g/L sodium azide, by agitating at room temperature for 2 hours. Then the mixture was centrifuged at 1000g for 10 minutes. The supernatant was collected for cytokine assay using a related cytokine detection ELISA kit according to the manufacturer’s instructions.

### Cell staining and flow cytometry

Mouse primary cells isolated from spleens, thymus, and lungs were stained with fluorescent antibodies and analyzed by flow cytometry.

For experiments with live/dead criteria, cells were first stained with Fixable Viability Dyes (FVD) (Thermo Fisher Scientific) according to the manufacturer’s instructions. Subsequent surface marker staining was performed in the Flow Cytometry Staining Buffer (Thermo Fisher Scientific). Intracellular staining for flow panels containing nuclear proteins was performed using the eBioscience FoxP3/Transcription Factor Staining Buffer Set (Thermo Fisher Scientific) according to the manufacturer’s instructions. For intracellular staining of cytoplasmic proteins, the Fixation/Permeabilization Solution Kit (BD Biosciences) was used according to the manufacturer’s instructions. For PE-conjugated influenza A NP_311-325_/I-Ab tetramer staining, incubate for 120 minutes at 37°C in the dark. For influenza A PA_224-233_/Db tetramer staining, incubate for 120 minutes at 4°C. For phospho antibody staining, cells were incubated with FVD (cell viability dye) for 15 minutes at room temperature in a tube, re-suspended with 200 μL pre-warmed 1×BD Phosflow Lyse/Fix buffer directly into the tube, and incubated at 37° for 10-15 minutes, followed by centrifugation at 300×g for 5 minutes. The resulting cell pellets were washed once with FACS buffer, permeabilized with 200 μL of BD Phosflow Perm Buffer III for 45 minutes on ice, and centrifuged at 300×g for 5 minutes. The cell pellets were washed again with FACS buffer, centrifuged at 300×g for 5 minutes, and incubated with antibodies in FACS buffer for 45 minutes-1 hour at room temperature.

After gating live CD45^+^ cells, CD4^+^ T cells are gated as CD3^+^CD4^+^CD8^−^, CD8^+^ T cells are gated as CD3^+^CD8^+^CD4^−^, NK cells are gated as CD3^−^NK1.1^+^, B cells are gated as B220^+^CD19^+^, macrophages are gated as F4/80^+^CD11b^+^Ly6G^−^, monocytes are gated as CD11b^+^Ly6C^+^Ly6G^−^F4/80^low^, DCs are gated as CD11c^+^MHC-II^+^, and neutrophils are gated as CD11b^+^Ly6G^+^Ly6C^int^. For the thymus, after gating live CD45^+^ cells, DN (Double-Negative) are gated as CD4^−^CD8^−^, DP (Double-Positive) are gated as CD4^+^CD8^+^, CD4 Single-Positive (SP) are gated as CD4^+^CD8^−^, CD8 Single-Positive (SP) are gated as CD4^−^CD8^+^.

Data was collected on BD LSR-Fortessa 4-15 flow cytometer, Cytek Aurora, or Attune NxT 4-14 and analyzed using FlowJo v10.4. A complete list of antibodies used for flow cytometry in this study is provided in Data S13.

### Milk microbiome 16S sequencing

After collection of milk (batch 1: n=3 per group; batch 2: n=5 per group. Total n = 8 per group) and pup fecal samples (n = 9 per group), 50 μL of each milk sample was mixed with 150 μL DNA/RNA Shield^™^ (Zymo Research, R1100-250), and 20 mg of each fecal sample was mixed with 200 μL DNA/RNA Shield^™^ (Zymo Research, R1100-250). Samples were then shipped to Zymo Research (Irvine, CA) for 16S rRNA gene sequencing to profile the microbiome. In brief, the ZymoBIOMICS^®^-96 MagBead DNA Kit (Zymo Research) was used to extract DNA using an automated platform. Bacterial 16S ribosomal RNA gene-targeted sequencing was performed using the Quick-16S^™^ NGS Library Prep Kit (Zymo Research). The bacterial 16S primers amplified the V3-V4 region of the 16S rRNA gene. The sequencing library was prepared using an innovative library preparation process in which PCR reactions were performed in real-time PCR machines to control cycles and, therefore, limit PCR chimera formation. The final PCR products were quantified with qPCR fluorescence readings and pooled together based on equal molarity. The final pooled library was cleaned with the Select-a-Size DNA Clean & Concentrator^™^ (Zymo Research), then quantified with TapeStation^®^ (Agilent Technologies) and Qubit^®^ (Thermo Fisher Scientific). The ZymoBIOMICS^®^ Microbial Community Standard (Zymo Research) was used as a positive control for each DNA extraction, if performed. The final library was sequenced on Illumina^®^ MiSeq^™^ with a v3 reagent kit (600 cycles). The sequencing was performed with 10% PhiX spike-in. For Bioinformatics Analysis, unique amplicon sequence variants were inferred from raw reads using the DADA2 pipeline(42). Potential sequencing errors and chimeric sequences were also removed with the Dada2 pipeline. Chimeric sequences were also removed with the DADA2 pipeline. Taxonomy assignment was performed using Uclust from Qiime v.1.9.1 with the Zymo Research Database, a 16S database that is internally designed and curated, as a reference. Composition visualization, alpha-diversity, and beta-diversity analyses were performed with Qiime v.1.9.1(43).

### Neonatal *Salmonella enterica serovar Typhimurium* challenge

C57BL/6J neonatal mice from the CON or TVA groups were challenged with *Salmonella enterica serovar* Typhimurium (*S. Typhimurium*, provided by Eugene B. Chang’s lab). Two-week-old pups from either group were treated with 1×10^7^ CFU *S. Typhimurium* in 100 μL PBS *via* oral gavage. BW was recorded immediately prior to challenge (day 0) and again on day 3 post-challenge. On day 3, mice were euthanized, and the liver and spleen were collected for bacterial burden quantification; spleens were additionally processed for flow cytometric analyses. Bacterial burden was determined by plating tissue homogenates and enumerating colony-forming units (CFU). Briefly, freshly collected spleen or liver was weighed, transferred to a tube containing 500 μL PBS, and homogenized using a tissue grinder and pestle. Homogenates were serially diluted 10-fold in PBS, and 5 μL of each dilution was plated in duplicate or triplicate on LB agar. Plates were incubated aerobically at room temperature for 12 h, and colonies were counted to calculate CFU per tissue (optionally normalized to tissue weight). Single-cell suspensions were prepared from spleens for flow cytometry. Panels included leukocyte lineage markers (CD45, CD3, CD4, CD8, TCRδ, NK1.1, CD11b, Ly6G, F4/80, Ly6C, MHC II, CD11c, B220) and Th1-response markers (T-bet, IFN-γ). For intracellular cytokine staining, splenocytes were stimulated *ex vivo* with PMA in the presence of brefeldin A for 6 h, followed by intracellular detection of IFN-γ and T-bet in CD4^+^ T cells and IFN-γ in CD8^+^ T cells.

### LIME-seq and data analysis

Total RNA was isolated from stool samples using TRIzol following standard protocols and subjected to LIME-seq library preparation as previously described(19). Briefly, RNA was fragmented before end-repaired, and ligated to 3′ adapters, followed by reverse transcription using a robust HIV reverse transcriptase to enable efficient readthrough at methylated nucleotides. cDNA products were subsequently ligated to adapters, PCR-amplified, and sequenced on an Illumina NovaSeq platform (PE150). Only R1 reads have been applied for the downstream analysis.

LIME-seq data analysis was performed as previously described(19). Sequencing reads were first aligned to the mouse genome (GRCm38) to remove host-derived RNA, and the remaining reads were classified using Kraken2 standard database to estimate microbiome abundance at the genus level. Mutation signatures detected by LIME-seq enable sensitive profiling of multiple RNA modifications and are correlated with microbial metabolic activity(44–48). We further examined microbiome-associated methylation patterns across diverse taxa. Although differential analyses identified candidate differences between groups, quantile–quantile (QQ) plots did not support strong statistically significant or group-specific methylation changes, indicating largely preserved microbiome modification landscapes between conditions.

### TVA treatment for human neonatal naïve CD4^+^ T cells

Three independent human umbilical cord blood samples were purchased from BioIVT. Naïve CD4^+^ T cells were isolated using the EasySep^™^ Human Naïve CD4^+^ T Cell Isolation Kit (STEMCELL Technologies) according to the manufacturer’s instructions. Purified cells were treated with vehicle (DMSO) or TVA (20 μM or 100 μM) for 24 h, followed by the addition of a Th1 polarization cocktail for 48 h or 72 h. Cells were then analyzed by flow cytometry to quantify the frequencies of T-bet^+^ and T-bet^+^IFN-γ^+^ cells within the CD4^+^ T cell population.

### Quantitative real-time PCR (RT-PCR)

Total RNA was extracted with TRIzol Reagent (Invitrogen) and then used for synthesizing the first-strand cDNA with PrimeScript^™^ 1st strand cDNA Synthesis Kit (Takara) according to the manufacturer’s instructions. Quantitative RT-PCR was conducted with iTaq Universal SYBR Green Supermix (Bio-Rad).

### RNA interference (RNAi) with Accell siRNA

Neonatal mouse primary T cells were isolated and cultured in replete media (RPMI 1640 medium or Click’s medium containing 10% FBS, 55 μM 2-mercaptoethanol, 2 mM glutamine, penicillin/streptomycin, and either PHA, CD3 or IL-2) for 24 hours, followed by incubation with Accell delivery mix (Accell siRNA Delivery Media (Horizon Discovery) with 1 μM siRNA, 20 IU/mL IL-2 and 1% FBS) for 72 hours. Cells were collected for subsequent function analysis as well as depletion efficiency validation using RT-PCR.

### Quantification and statistical analysis

All statistical analyses were performed using GraphPad Prism 9. Sample sizes were determined a priori on the basis of statistical power considerations and feasibility; unless otherwise stated, experiments were designed to provide approximately 80–90% power to detect biologically meaningful effects at a two-sided significance level of 0.05, using effect sizes and variability estimated from pilot experiments and/or prior experience with the models used, and planned analyses accounted for multiple comparisons where applicable. Animals and samples were assigned to experimental groups without regard to outcome, and measurements were collected with investigators blinded to group identity when feasible. All reported n values represent independent biological replicates (independent animals or independently prepared samples); technical replicates were used to ensure measurement reliability and were averaged within each independent experiment before statistical testing. Data normality was assessed using the Shapiro–Wilk test (n ≤ 50) or the D’Agostino–Pearson omnibus test (n > 50), with Q–Q plot inspection, and variance homogeneity was tested using the Brown–Forsythe (Levene) test. Parametric tests (two-tailed Student’s t-test or one-way ANOVA) were used only when these assumptions were satisfied; otherwise, Welch’s corrections or nonparametric tests (Mann–Whitney U or Kruskal–Wallis with Dunn’s post hoc) were applied. Exact sample sizes, statistical tests, and any multiple-comparison procedures are reported in the figure legends. All data are representative of at least two to three independent experiments, and all attempts at replication were successful.

## Supplementary Material

aea4041_Suppl Excel_seq1_v2

aea4041_Suppl Excel_seq2_v2

aea4041_Suppl Excel_seq5_v2

aea4041_Suppl Excel_seq6_v2

aea4041_Suppl Excel_seq3_v2

aea4041_Suppl Excel_seq4_v2

aea4041_Suppl Excel_seq7_v2

aea4041_Suppl Excel_seq8_v1

aea4041_Suppl Excel_seq9_v1

aea4041_Suppl Excel_seq10_v3

aea4041_Suppl Excel_seq11_v1

aea4041_Suppl Excel_seq12_v1

aea4041_Suppl Excel_seq13_v2

Supplementary Materials_final version

## Figures and Tables

**Fig. 1.**
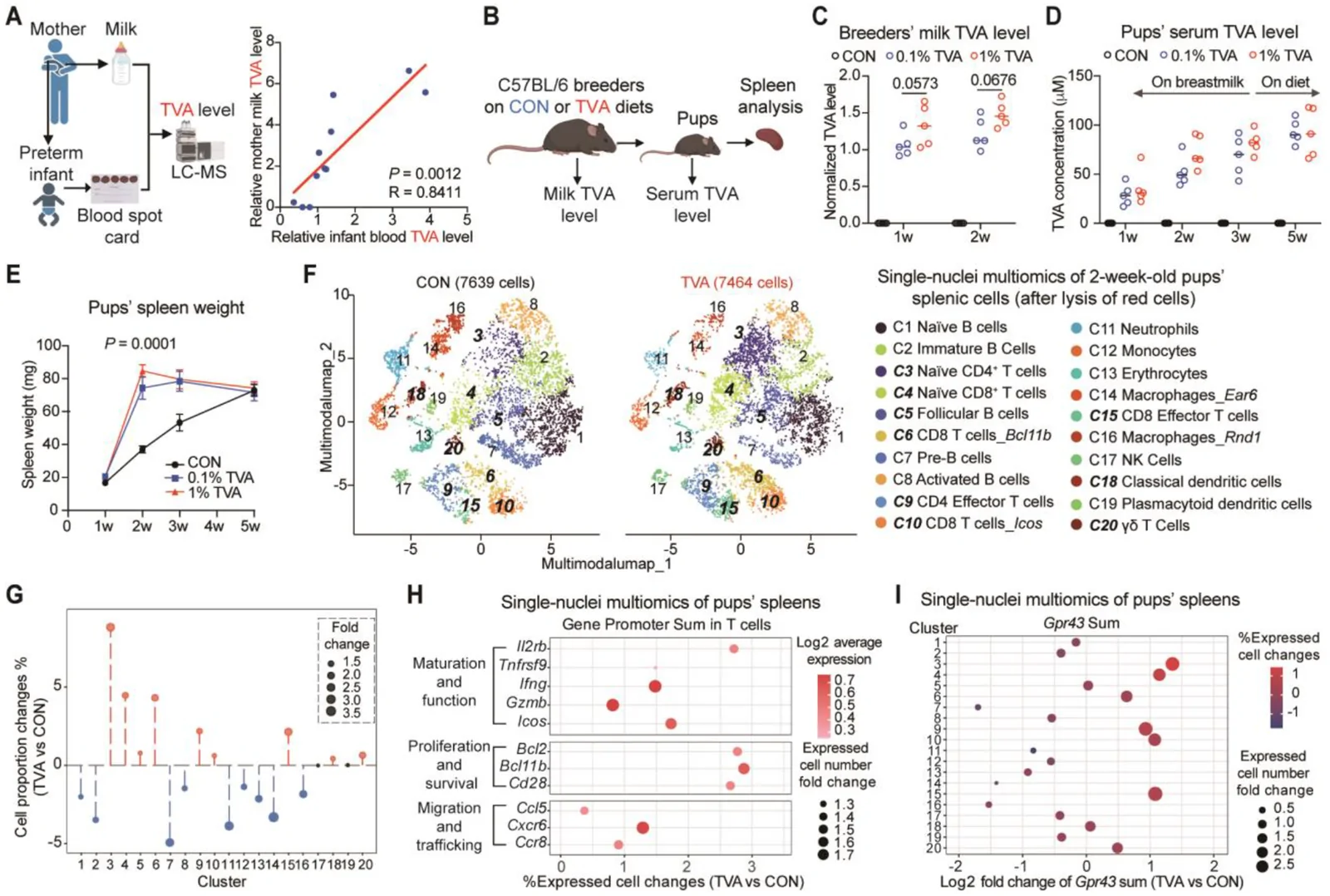
Maternal TVA reshapes neonatal T cell development *via* breastfeeding. (**A**) *Left*, schematic for mother–preterm infant pairs. Human breastmilk and paired infant blood spot cards were used to quantify TVA. *Right*, correlation between relative maternal milk TVA and relative infant blood TVA; each dot represents one mother–infant pair (*n*=11). Red line, linear regression. (**B**) Experimental design: feeding mouse breeders with TVA or CON diets. (**C**) Normalized TVA levels in breastmilk from breeders on CON, 0.1% or 1% TVA diet when their pups are at 1 or 2 weeks old (*n*=5). The TVA level of the 1-week 0.1% TVA group was normalized to one; other samples were relative to this reference. (**D**) TVA levels in pups’ serum (*n* = 5). Shading, the TVA exposure window. (**E**) Effects of TVA-milk on pups’ spleen weight (1w: *n*=4 CON, 4 0.1%TVA, 14 1% TVA; 2w: *n*=8, 8, 3; 3w: *n*=14, 5, 5; 5w: *n*=11, 8, 8). (**F**) TVA-milk’s effects on single-nuclei multi-omics of 2-week-old pups’ splenic cells (numbers indicate total nuclei). UMAP shows identified cell subsets. (**G**) Cell proportion changes of different subsets in (F), point size denotes the magnitude of change, with positive and negative values indicating relative increase or decrease in the TVA group. (**H**) Gene promoter sum scores change between TVA and CON groups in (F). Highlighting genes linked to T cell maturation/function, proliferation/survival, and migration/trafficking. Dot color indicates average signal; dot size indicates changes in the frequency of positive cells. (**I**) Cluster-level changes in *Gpr43* sum between TVA and CON groups in (F). Dot color, changes in the %expressed cell number; dot size, fold change of the expressed cell number. Data are mean (C, D) or mean ± SEM (E); symbols indicate individual mice. (C) and (D) are representative of 3-4 independent experiments with five mice per group; (E) is representative of ≥3 independent experiments with ≥3 samples per group. In (F), samples were collected from 3-5 independent experiments, with five mouse samples pooled per group. In (A), Pearson correlation analysis was used, and shading indicates the 95% confidence interval. Statistical significance was determined by using two-way ANOVA and Holm-Sidak post-hoc tests for (C) and (E).

**Fig. 2.**
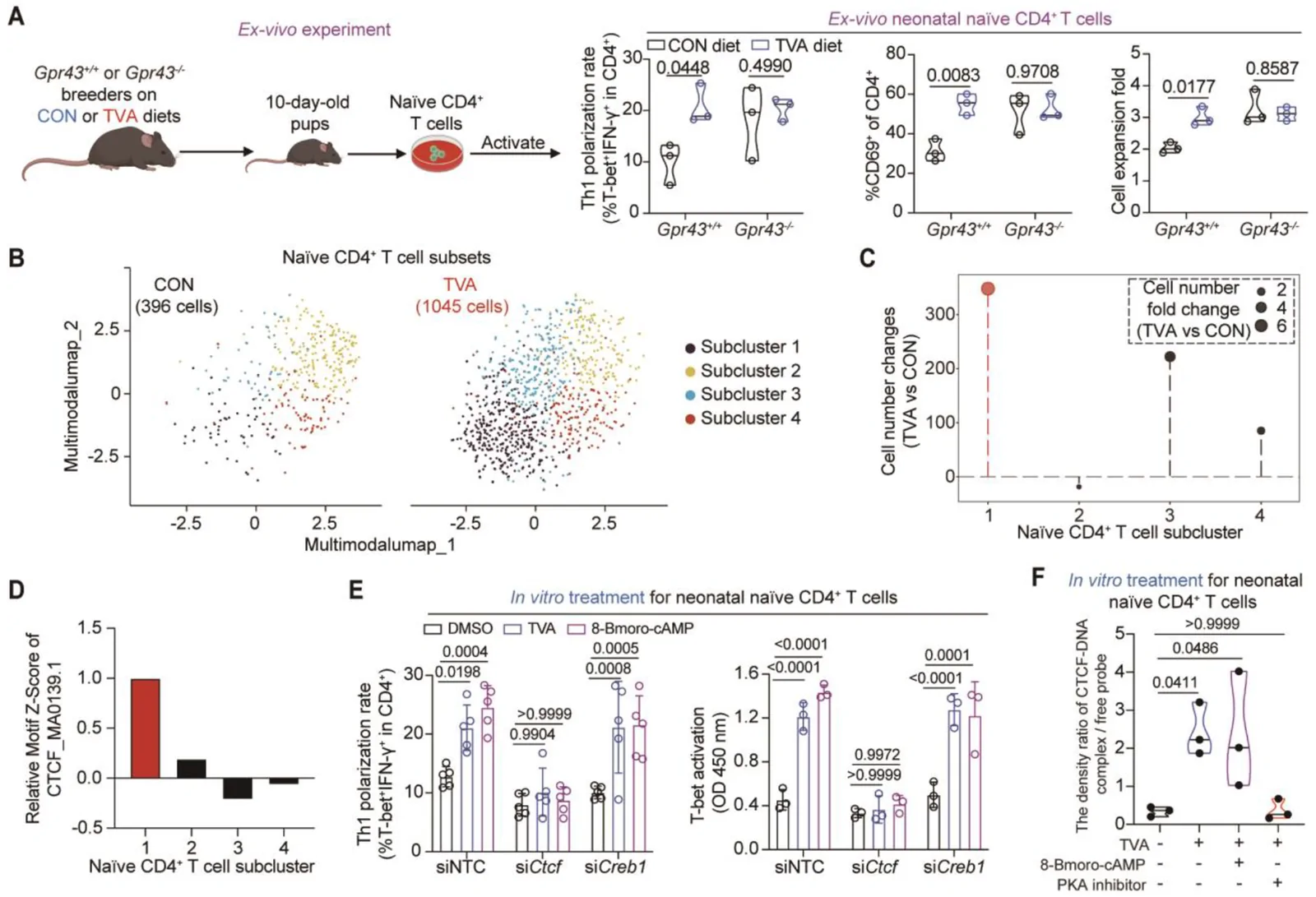
TVA-milk reprograms neonatal naïve CD4^+^ T cells through a GPR43- CTCF axis. (**A**) Schematic of *ex-vivo* experiments to examine neonatal naïve CD4^+^ T cells from the offspring of WT (*Gpr43*^+/+^) or *Gpr43* knockout (*Gpr43*^−/−^) breeders fed on CON or TVA diet (*left*). Following naïve CD4^+^ T cell activation or stimulation, changes in Th1 polarization, %CD69^+^, and cell expansion are shown (*right*) (*n*=3 each). (**B**) Integrated single-nucleus multi-omics UMAP of neonatal naïve CD4^+^ T cells (cluster 3 in Fig. 1F), showing four transcriptionally distinct subclusters; numbers indicate total nuclei. (**C**) Cell number changes (TVA vs. CON) of different naïve CD4^+^ T cell subsets in 2-week-old pups’ splenic cells. Points indicate the change in cell number for each subcluster; point size denotes the fold change in cell number. (**D**) Relative CTCF motif activity (CTCF_MA0139.1 motif Z-score) across the four naïve CD4^+^ T cell subclusters in 2-week-old pups’ splenic cells. (**E**) Changes in Th1 polarization (*n*=5 each), and T-bet activity (OD450) (*n*=3 each) following *in vitro* treatment of neonatal naïve CD4^+^ T cells with DMSO, TVA, or 8-Bromo-cAMP, under siNTC, si*Ctcf*, or si*Creb1* conditions. (**F**) Changes in CTCF activity, measured by a probe-based DNA-binding assay (CTCF–DNA complex/free probe ratio), in neonatal naïve CD4^+^ T cells treated with TVA in the presence or absence of 8-Bromo-cAMP or PKA inhibitor (*n*=3 each). In (A) and (F), violin plots depict the distribution with central tendency indicated; symbols indicate individual mice; data are representative of 2-3 independent experiments with three mice per group. In (E), error bars indicate the mean ± SD; symbols indicate individual mice; data are representative of 2-4 independent experiments with 3-5 mice per group. For (A), (E), and (F), statistical significance was determined by using two-way ANOVA and Holm-Sidak post-hoc tests.

**Fig. 3.**
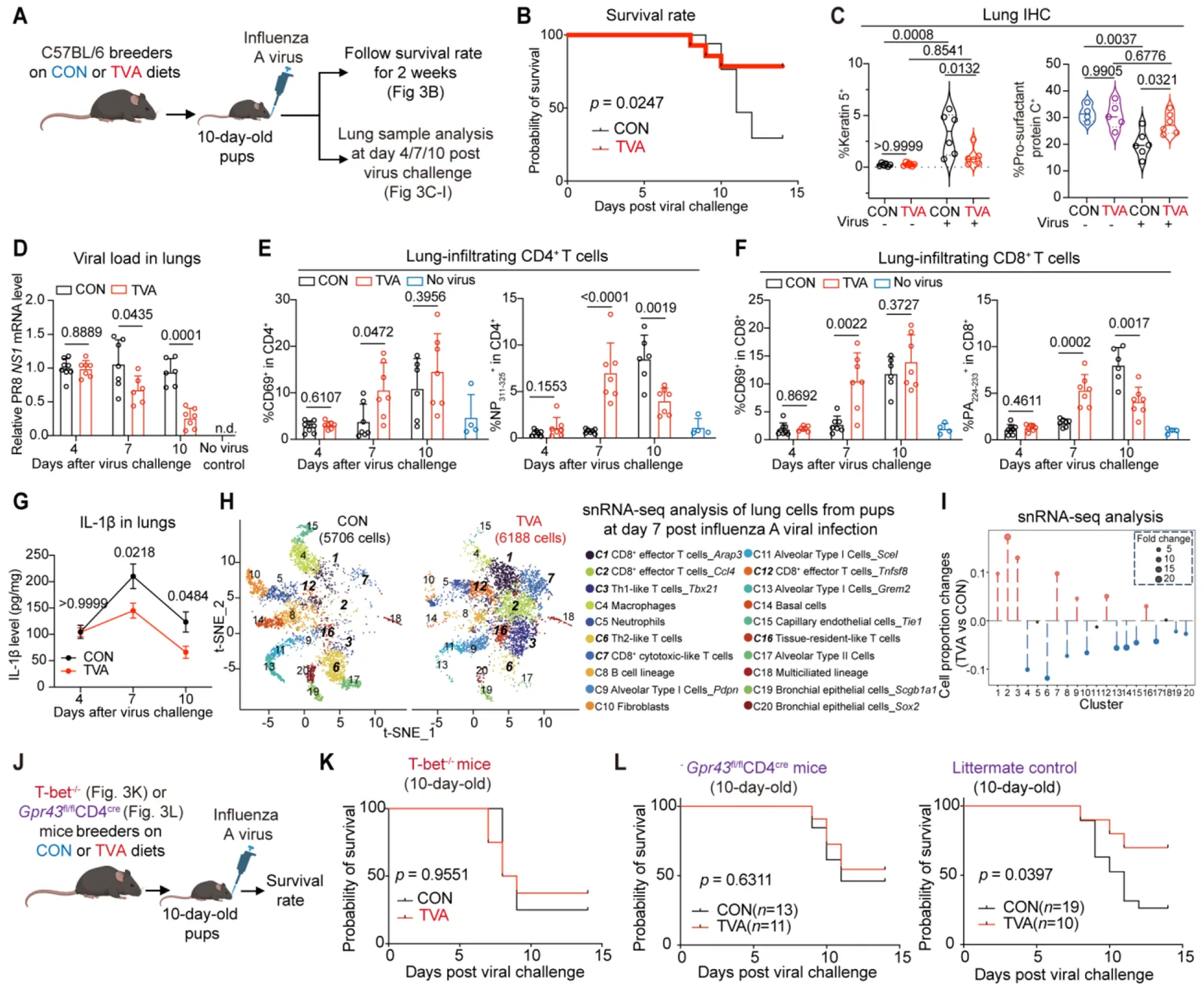
TVA-milk programs neonatal T cells for anti-viral responses. (**A**) Schematic of influenza A virus (PR8) challenge for neonatal mice with/without TVA in breastmilk. (**B**) Survival rate of (A) (*n*=17 CON, 14 TVA). (**C**) Effects of TVA-milk on lung epithelial injury at day 7 after infection, assessed by keratin 5 staining, and on normal epithelium, assessed by pro–surfactant protein C staining. Summary data are shown (no virus: *n*=4 CON, 5 TVA; virus: *n*=6/group). (**D**) Lung viral burden in infected pups, measured by influenza PR8 *NS1* mRNA (n.d., not detected) (day 4: *n*=8 CON, 7 TVA; day 7: *n*=7, 6; day 10: *n*=6, 7). (**E**) Effects of TVA-milk on lung-infiltrating neonatal CD4^+^ T cell activation (CD69, *left*) and virus-recognizing activity (NP_331-325_-specific, *right*). “No virus”, uninfected controls. (D4: *n*=8, 7; D7: *n*=7, 7; D10: *n*=6, 7; no virus *n*=4). (**F**) Effects of TVA-milk on lung-infiltrating neonatal CD8^+^ T cell activation (CD69, *left*) and virus-recognizing activity (PA_224-233_-specific, *right*). Group sizes are as in E. (**G**) Lung IL-1β levels after infection (*n*=5). (**H**) SnRNA-seq analysis of lung cells of pups at day 7 post PR8 infection in CON and TVA groups (numbers indicate total nuclei). t-SNE displaying different cell subsets. (**I**) Cell proportion changes (TVA vs. CON) of different subsets in (H). Point size denotes the fold change. (**J**) Schematic of PR8 challenge for neonatal T-bet^−/−^ or *Gpr43*^fl/fl^CD4^cre^ mice with/without TVA-breastmilk. (**K**) Survival of infected T-bet^−/−^ pups (*n*=4 CON, 8 TVA). (**L**) Survival of infected *Gpr43*^fl/fl^CD4^cre^ pups (*left*; *n*=13 CON, 11 TVA) or littermate controls (*right*; *n*=19 CON, 10 TVA). Data in (B), (K), and (L) are Kaplan-Meier survival curves and are representative of 3-5 independent experiments with ≥10 mice per group (B, L) or ≥4 mice per group (K). Symbols indicate individual mice (C to F). In C, violin plots show distributions with overlaid summary statistics. In (D) to (F), error bars indicate the mean ± SD; data are representative of 3-4 independent experiments with ≥4 mice per group. In (G), error bars indicate the mean ± SEM; data are representative of 3-4 independent experiments with five mice per group. Statistical significance was determined by using the log-rank (Mantel-Cox) test for (B), (K), and (L), two-way ANOVA and Holm-Sidak post-hoc tests for (C) to (G).

**Fig. 4.**
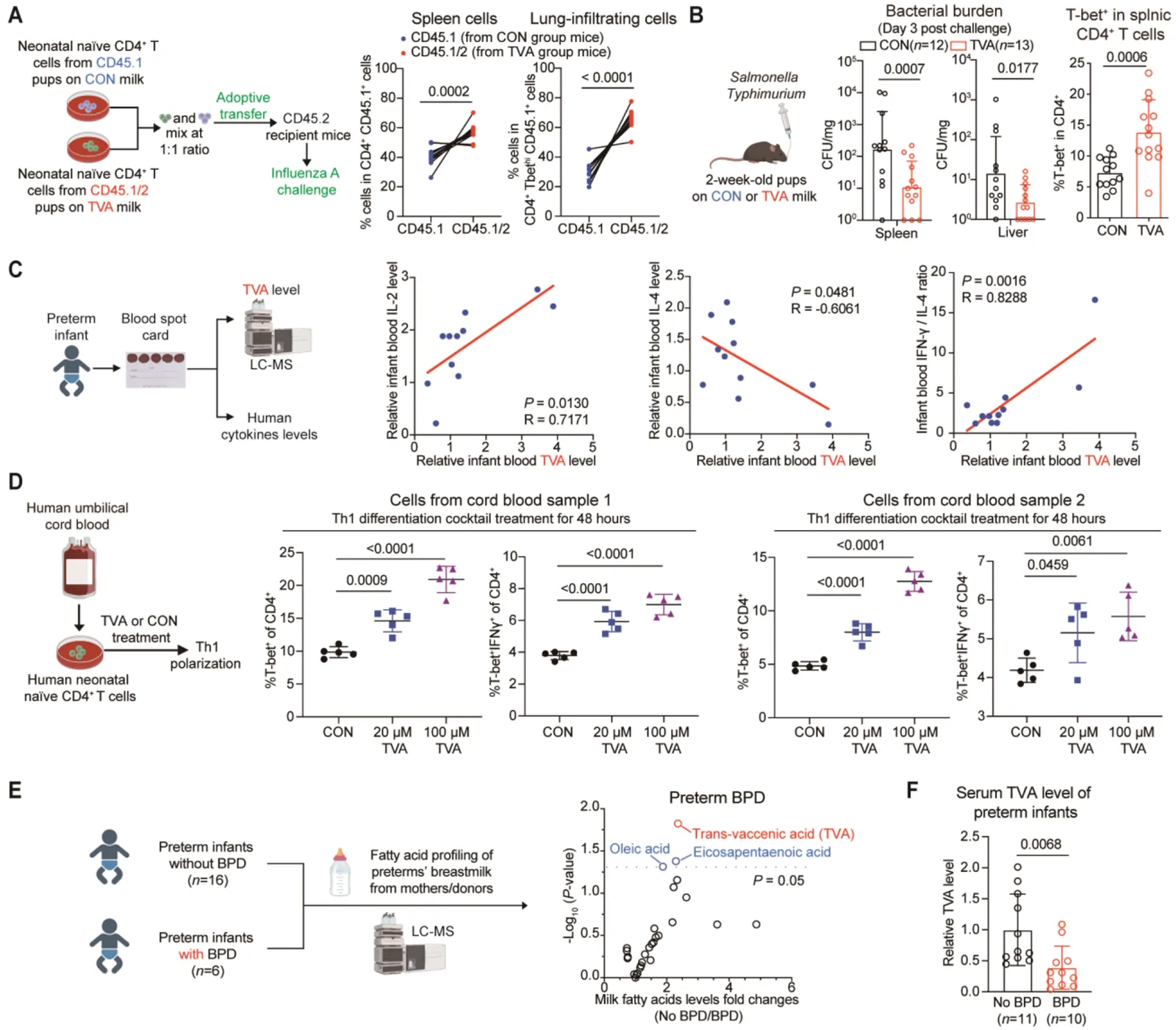
TVA promotes neonatal Th1 programming. (**A**) *Left*, adoptive-transfer strategy comparing neonatal naïve CD4^+^ T cells exposed to CON or TVA milk. *Middle* and *right*, relative contribution of each donor population among splenic CD4^+^ cells and lung-infiltrating CD4^+^T-bet^+^ cells, respectively (*n*=13 recipients). (**B**) *Left*, schematic of *Salmonella Typhimurium* challenge in 2-week-old pups exposed to TVA or CON milk. *Middle*, bacterial burden in spleen and liver. *Right*, frequency of T-bet^+^ cells among splenic CD4^+^ T cells at day 3 (*n*=12 CON, 13 TVA). (**C**) *Left*, schematic for measurement of TVA and cytokines in preterm infants’ blood spot cards. *Right*, correlations between TVA and cytokine levels in preterm infant samples (*n*=11), red lines indicate linear regression. (**D**) *Left*, schematic for testing the effects of TVA on Th1 polarization of human neonatal naïve CD4^+^ T cells isolated from cord blood. *Middle* and *right*, Th1 polarization (T-bet^+^ or T-bet^+^IFN-γ^+^) after TVA treatment. (**E**) *Left*, schematic for fatty acid profiling of breastmilk from mothers of preterm infants with/without bronchopulmonary dysplasia (BPD). *Right*, volcano plot of differential fatty acid abundance between no-BPD and BPD groups. Dotted line, nominal significance threshold (*P*=0.05) (no BPD, *n*=16; BPD, *n*=6). (**F**) Relative serum TVA levels in preterm infants (no BPD *n*=11, BPD *n*=10). Symbols indicate individual recipient mice in (A); individual mice in (B); individual infant in (C) and (F); technical replicates of each human sample in (D). In (A), the line pairs the CD45.1^+^ and CD45.1/2^+^ cells in the same recipient; data are representative of four independent experiments with more than 38 donor mice per group. In (B), data are mean ± SD and are representative of three independent experiments with ≥12 mice per group. In (D), data are mean ± SD; each human sample is representative of one independent experiment (3 human samples in total, including fig. S14). In (F), data are mean ± SD. Statistical significance was determined by using a paired two-tailed Student’s t-test for (A), an unpaired two-tailed Student’s t-test for (B), (E), and (F). For (C), the correlation coefficient (r) and *P*-value were determined using Pearson correlation analysis; the shaded area represents the 95% confidence interval. One-way ANOVA and Tukey post-hoc tests for (D).

**Fig. 5.**
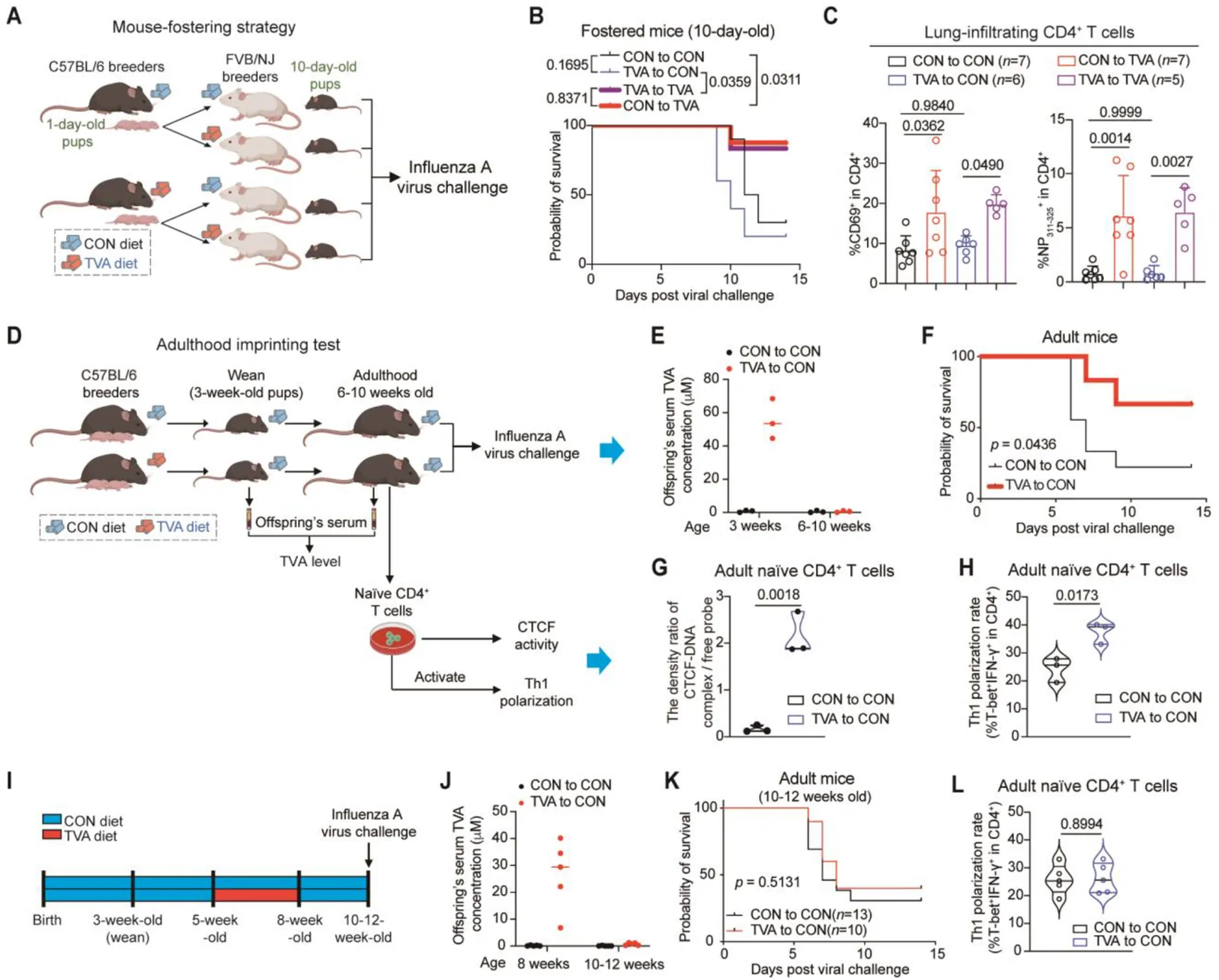
Early-life exposure to TVA *via* breastfeeding impacts immune imprinting. (**A**) Strategy used to test whether TVA protects neonatal mice from PR8 infection through prenatal and/or postnatal exposure. (**B**) Survival of (A) (*n*=10 CON to CON, 8 CON to TVA, 5 TVA to CON, 6 TVA to TVA). (**C**) Effects of prenatal and lactational TVA exposure on lung-infiltrating neonatal CD4^+^ T cell activation (%CD69^+^) and virus recognition (NP_331–325_^+^) (*n*=7, 7, 6, 5). (**D**) Experimental design to test immune imprinting by TVA-milk. Breeders were fed CON/TVA diets; offspring were switched to the CON diet after weaning and challenged with PR8 as adults. (**E**) Serum TVA concentrations in offspring at weaning and adulthood (*n*=3). (**F**) Survival after PR8 infection of 6-10-week-old mice exposed to TVA-enriched or control milk (*n*=9 CON to CON, 7 TVA to CON). (**G-H**) *Ex vivo* CTCF activity (G) and Th1 polarization (H) of naïve CD4^+^ T cells from adult mice exposed to TVA-enriched or control milk (*n*=3). (**I**) Experimental design in which TVA exposure was restricted to 5-8-week-old mice to test whether later exposure induces immune imprinting detectable in 10-12 weeks of age. (**J**) Serum TVA concentrations at 8 weeks and 10-12 weeks after TVA diet exposure from 5-8 weeks of age (*n*=5). (**K**) Survival after PR8 infection of 10-12-week-old mice fed TVA/CON diet from 5-8 weeks of age (*n*=13, 10). (**L**) Th1 polarization of naïve CD4^+^ T cells in 10-12-week-old mice fed the TVA/CON diet from 5-8 weeks of age (*n*=5). Symbols indicate individual mice in (C), (E), (G-H), (J), and (L). (B), (F), and (K) are Kaplan-Meier overall survival curves and are representative of 3-4 independent experiments, with ≥5 mice per group for (B) and ≥10 mice per group for (F) and (K). (C) shows mean ± SD and is representative of 2-3 independent experiments with ≥5 mice per group. In (E) and (J), lines indicate the mean; data are representative of 2-3 independent experiments with ≥3 mice per group. In (G), (H), and (L), violin plots show the distribution with overlaid summary statistics; data are representative of three independent experiments with five mice per group. *P* values were determined by the log-rank (Mantel-Cox) test for (B), (F), and (K), one-way ANOVA with Tukey’s post hoc test for (C), and unpaired two-tailed Student’s t-test for (G), (H), and (L).

## Data Availability

16S amplicon sequencing data have been deposited in the NCBI Sequence Read Archive (SRA) repository with the accession numbers PRJNA1220719, PRJNA1402979, and PRJNA1403008. The spleen Single Cell Multiome ATAC + Gene Expression data have been deposited in the GEO repository with the accession number GSE289161. The lung Single Cell Multiome ATAC + Gene Expression data have been deposited in the GEO repository with the accession number GSE289160. The pups’ fecal sample RNA LIME-seq data have been deposited in the GEO repository with the accession number GSE314181. All other data needed to evaluate the conclusions in the paper are present in the paper or the Supplementary Materials. No new code or materials were made in the study.
