# Supplementary material for: Maternal trans-vaccenic acid shapes neonatal T cell development and early-life immune imprinting
Source: Science. Author manuscript; Available in PMC 2026 Sep 29. (PMC13621437; doi:10.1126/science.aea4041)
Supplement: Supplementary file 1 [file NIHMS2210149-supplement-Supplementary_Materials_final_version.docx]

Supplementary Materials for

**Maternal trans-vaccenic acid shapes neonatal T cell development and early-life immune imprinting**

Hao Fan *et al*.

Corresponding author: eclaud@bsd.uchicago.edu; chuanhe@uchicago.edu; jingchen@uchicago.edu

**The PDF file includes:**

Figs. S1 to S17

Captions for Data S1 to S13

**Other Supplementary Material for this manuscript includes the following:**

MDAR Reproducibility Checklist

Data S1 to S13

**Fig. S1.** **TVA-milk’s effects on the immune system of mouse pups.** **(A)** Effects of TVA-milk on pups’ body weight. **(B)** TVA-milk’s effects on immune cell number of 2-week-old pups’ spleens (splenocyte, CD4^+^, CD8^+^, B: n=14 each; neutrophil, DC, monocyte: n=8 each). **(C)** TVA-milk’s effects on immune cell percentage of splenocytes of 2-week-old pups’ spleens (splenocyte, CD4^+^, CD8^+^, B: n=14 each; neutrophil, DC, monocyte: n=8 each). **(D)** TVA-milk’s effects on immune cell number of 5-week-old pups’ spleens (n=11 CON, n=8 TVA). **(E)** TVA-milk’s effects on the immune cell percentage of splenocytes of 5-week-old pups’ spleens (n=11 CON, n=8 TVA). **(F-G)** Effects of TVA-milk on pups’ splenic CD4^+^ (**F**) or CD8^+^ (**G**) T cell numbers at 1, 2, 3, and 5 weeks old (1w: n=4 CON, n=4 TVA; 2w: n=14 CON, n=14 TVA; 3w: n=5 CON, n=6 TVA; 5w: n=11 CON, n=8 TVA). **(H)** Effects of TVA-milk on pups’ spleen naïve, central memory, and effector memory CD4^+^ T cells at 2 weeks old (n=14 each). **(I)** Effects of TVA-milk on pups’ splenic Treg cells (Foxp3^+^CD25^hi^ in CD4^+^ T cells) at 2 weeks old (n=8 CON, n=3 TVA). **(J)** Effects of TVA-milk on pups’ splenic naïve, central memory, and effector memory CD8^+^ T cells at 2 weeks old (n=14 each). **(K)** Effects of TVA-milk on pups’ splenic %CD69^+^ of CD4^+^ or CD8^+^ T cells at 2 weeks old (n=14 each). **(L)** Effects of TVA-milk on pups’ thymus weight at 2 weeks old (n=16 CON, n=19 TVA). **(M)** Effects of TVA-milk on the thymic development of pups at two weeks old, including the percentages of double-negative (DN), double-positive (DP), CD4 single-positive (4SP), and CD8 single-positive (8SP) thymocytes (n=14 each). **(N)** TVA-milk’s effects on immune cell number of 2-week-old pups’ lungs (n=8 each). **(O)** Effects of TVA-milk on pups’ lung naïve T cells at 2 weeks old (n=4 each). **(P)** Effects of TVA-milk on pups’ lung %CD69^+^ of CD4^+^ or CD8^+^ T cells at 2 weeks old (n=4 each). **(Q)** Effects of TVA-milk on pups’ lung Treg cells (Foxp3^+^CD25^hi^ in CD4^+^ T cells) at 2 weeks old (n=4 each). Symbols indicate individual mice for (A) to (Q). In (A), violin plots depict the distribution with central tendency indicated; data are representative of 3-5 independent experiments with ≥ 6 mice per group. In (B) to (H), error bars indicate the mean ± SD; data are representative of 3-5 independent experiments with ≥ 4 mice per group. In (I), error bars indicate the mean ± SD; data are representative of two independent experiments with ≥ 3 mice per group. In (J) to (M), error bars indicate the mean ± SD; data are representative of 3-5 independent experiments with ≥ 14 mice per group. In (N) to (Q), error bars indicate the mean ± SD; data are representative of two independent experiments with four mice per group. Statistical significance was determined by using two-way ANOVA and Holm-Sidak post-hoc tests for (A), (F), and (G), an unpaired two-tailed Student’s t-test for (B) to (E), and (H) to (Q).

**Fig. S2. The effects of TVA on the neonatal immune cells. (A)** Marker gene expression defines immune cell identities from splenic single-nuclei multiomics, the dot plot showing the expression of selected canonical marker genes across single-cell–defined clusters (C1–C20). Clusters were annotated as neutrophils, monocytes, macrophage subsets (Ear6⁺ and Rnd1⁺), classical and plasmacytoid dendritic cells, NK cells, γδ T cells, naïve and effector CD4⁺ and CD8⁺ T cell subsets, pre/immature/naïve/follicular/activated B cell subsets, and erythrocytes based on marker gene signatures. Dot size indicates the percentage of cells within each cluster expressing the indicated gene, and dot color denotes the average (scaled) expression level. **(B)** Each cell cluster’s CREB1_MA0018.4 motif changes between the TVA and CON group in 2-week-old pups’ splenic cells. Bubble plot summarizing cluster-level changes in CREB1_MA0018.4 motif signal between TVA and control conditions. Each dot represents one cluster (1–20). The x-axis shows the log2 fold change of CREB1_MA0018.4 motif Z-score (TVA vs CON). Dot color indicates the change in the percentage of motif-positive cells in each cluster (TVA vs CON), and dot size denotes the fold change of the expressed cell number (TVA vs CON). **(C)** Each cell cluster’s TBX21_MA0690.1 motif changes between the TVA and CON group in 2-week-old pups’ splenic cells. Bubble plot summarizing cluster-level changes in TBX21_MA0690.1 motif signal between TVA and control conditions. Each dot represents one cluster (1–20). The x-axis shows the log2 fold change of TBX21_MA0690.1 motif Z-score (TVA vs CON). Dot color indicates the change in the percentage of motif-positive cells in each cluster (TVA vs CON), and dot size denotes the fold change of the expressed cell number (TVA vs CON). **(D-F)** *Ex-vivo* experiments to examine neonatal naïve CD4⁺ T cells from the offspring of C57BL/6 or *Gpr43* knockout mouse breeders fed on either a CON or TVA diet. Following activation or stimulation, changes in Th1 (D), Th2 (E), and Th17 (F) polarization rate are shown. Representative plots show the frequencies of T-bet⁺IFN-γ⁺ cells (Th1; D), GATA3⁺IL-4⁺ cells (Th2; E), and RORγt⁺IL-17⁺ cells (Th17; F) within gated CD4⁺ T cells (percentages indicated). *Right*, summary quantification of Th2 and Th17 polarization (E–F); colors denote CON vs TVA groups, and *P* values are shown. For (E-F, *right*), violin plots depict the distribution with central tendency indicated; symbols indicate individual mice; data are representative of two independent experiments with three mice per group. Statistical significance was determined by using two-way ANOVA and Holm-Sidak post-hoc tests for (E), (F).

**Fig. S3. The effects of TVA on the neonatal naïve CD4^+^ T cells are mediated through a GPR43-cAMP-PKA-CTCF axis. (A)** Neonatal naïve CD4⁺ T cells (10–14-day-old pups) were treated in vitro with vehicle (DMSO) or the indicated fatty acids (OA, LA, PA, TVA, EA, and CLA) prior to Th1 polarization. Th1 output is shown as the frequency of T-bet⁺IFN-γ⁺ cells within CD4⁺ T cells; *P* values are indicated. (n=5 each) **(B)** Schematic depicting the design of *in-vitro* experiments to examine the effects of TVA on neonatal naïve CD4⁺ T cells from WT (*Gpr43*^+/+^) or *Gpr43* knockout (*Gpr43*^-/-^) neonatal mice (*left*). Following stimulation, changes in Th1 polarization rate (%T-bet⁺IFN-γ⁺ in CD4^+^) are shown (*right*) (n=3 each). The schematic figure was generated using BioRender. **(C)** Schematic depicting the design of *in-vitro* experiments to examine the effects of TVA on neonatal naïve CD4⁺ T cells with or without 8-Bmoro-cAMP, PKA inhibitor, CREB inhibitor (*left*). Following stimulation, changes in Th1 polarization rate (%T-bet⁺IFN-γ⁺ in CD4^+^) are shown (*right*) (n=4 each). The schematic figure was generated using BioRender. **(D)** Top 5 transcription factor motifs identified in each subcluster of naïve CD4⁺ T cells from splenic cells of 2-week-old pups following TVA-milk feeding. Dot color and size represent motif enrichment significance (−log₁₀ P) for the selected subcluster versus the other three subclusters (scale shown). **(E)** The motif score of CREB1 in different naïve CD4^+^ T cell subsets in 2-week-old pups’ splenic cells. **(F)** Relative promoter accessibility (promoter-sum scores) for ***Tbx21*** and ***Ifng*** across neonatal naïve CD4⁺ T cell subclusters. **(G)** Knockdown efficiency of ***Ctcf*** and ***Creb1*** in neonatal naïve CD4⁺ T cells following siRNA treatment, measured by qPCR (relative mRNA levels; **P values** shown) (n=5 each). **(H)** Representative chemiluminescent EMSA showing CTCF activity changes in neonatal CD4⁺ T cells following TVA treatment, with or without 8-Br-cAMP or PKA inhibitor. Positions of the shifted CTCF–DNA complex and free probe are marked. **(I)** Promoter sums of *Ctcf* in each of the subclusters of naïve CD4^+^ T cells in 2-week-old pups’ splenic cells. In (A) to (C), error bars indicate the mean ± SD; symbols indicate individual mice; data are representative of two independent experiments with five mice per group (A) or two independent experiments with ≥ 3 mice per group (B) and (C). For (G), bars indicate the mean; symbols indicate cells from individual mice; data are representative of two independent experiments with five mice per group. Statistical significance was determined by using one-way ANOVA and Tukey post-hoc tests for (A), two-way ANOVA and Holm-Sidak post-hoc tests for (B) and (C), and an unpaired two-tailed Student’s t-test for (G).

**Fig. S4. Dietary TVA for breeders has no significant effect on known immuno-molecules or microbiomes in the milk. (A-D)** Milk IgA (**A**), lactoferrin (**B**), IL-7 (**C**), and IL-1β (**D**) levels of TVA or CON diet-fed breeders (n=5 each). **(E)** Milk lipid profiling comparing the TVA versus CON groups. Volcano plot shows log₂ fold changes (TVA/CON) and corresponding significance (−log₁₀ P) (n=4); TVA is highlighted. The horizontal dashed line denotes the nominal *P* = 0.05 threshold. **(F)** Taxonomic composition of milk microbiota at the **order** level for batch 1 of the two independent sequencing batches (n=3). **(G)** Species-level taxonomic abundance heatmaps with unsupervised hierarchical clustering for batch 1 (n=3). **(H)** Taxonomic composition of milk microbiota at the **order** level for batch 2 (n=5). **(I)** Species-level taxonomic abundance heatmaps with unsupervised hierarchical clustering for batch 2 (n=5). In (A) to (D), violin plots show data distributions with summary statistics as indicated; each symbol represents an individual milk sample; data are representative of 3-4 independent experiments with five mice per group. For (E), the data are representative of 3-4 independent experiments with four mice per group. Statistical significance was determined by using an unpaired two-tailed Student’s t-test for (A) to (E).

**Fig. S5.** **TVA-milk’s effects on the fecal microbiomes of pups. (A–C)** Species-level taxonomic abundance heatmaps of fecal microbiota from pups in the CON and TVA groups with unsupervised hierarchical clustering. Samples are annotated by **diet group** and **litter identity** (symbols). (A) Fecal samples from pups born to breeders maintained long-term on the indicated diet. (B) Fecal samples from first-generation pups born to breeders that were switched to the indicated diet **3 days before delivery**. (C) Combined analysis including samples from both breeding paradigms. Color denotes relative taxon abundance (scale shown). **(D)** Principal component analysis (PCA) of microbiome abundance profiles derived from Low-Input Multiple Methylation Sequencing (LIME-seq) data of stool samples from control and treatment groups. PCA was performed using highly abundant microbial taxa, with abundance estimates obtained using Kraken2. **(E)** Differential microbiome abundance analysis based on LIME-seq data reveals minimal differences between control and treatment groups, indicating largely preserved microbial composition. **(F)** Quantile–quantile (QQ) plot of *p*-values from differential analysis of microbiome methylation ratios obtained by LIME-seq between control and treatment groups. The near-uniform distribution indicates limited statistically significant changes, suggesting that microbiome-associated methylation patterns, reflective of microbial metabolic states, remain largely unchanged between conditions. In (A) and (B), the data are representative of 3-6 independent experiments with nine mice per group. For (D) to (F) data are representative of two independent experiments with four mice per group.

**Fig. S6. TVA-milk ameliorates immune response to influenza A viral infection in neonatal mice.** **(A)** Body weight of pups in the TVA and CON group, prior to influenza A virus challenge (10-day-old) (n=17 CON, n=14 TVA). **(B)** Longitudinal body-weight trajectories following PR8 infection, presented as relative body weight over time for individual pups in the CON (n = 17) and TVA (n = 14) groups. The dashed blue line indicates the average trajectory of the uninfected (“no virus”) group. **(C)** Effects of TVA in breast milk on the clinical score of mice with lung infection by influenza A virus at 10-day-old (n=17 CON, n=14 TVA). Asterisks denote statistically significant differences between groups at the indicated time points. **(D)** Representative lung sections from CON and TVA groups, left uninfected (−) or infected with influenza A virus (+), stained by immunohistochemistry (IHC) for Keratin 5 (brown, top) to mark injury-associated epithelial remodeling and for pro–surfactant protein C (red, bottom) to mark normal alveolar epithelium. Scale bars, 1 mm. In (A), error bars indicate the mean ± SD; symbols indicate individual mice. In (C), error bars indicate the mean ± SEM. For (A) to (C), the data are representative of 3-5 independent experiments with fourteen mice per group. Statistical significance was determined by using an unpaired two-tailed Student’s t-test for (A), two-way ANOVA and Holm-Sidak post-hoc tests for (C).

**Fig. S7. TVA-milk promotes T cell responses to lung viral infection. (A)** Gating strategies of lung-infiltrating cells. **(B-D)**, Flow cytometric quantification shows the effects of TVA-milk on lung-infiltrating neonatal CD4^+^ T cell population (B), %CD44^+^ of CD4^+^ (C), %CD11a^+^CD49d^+^ of CD4^+^ (D) at the indicated days post-infection. “No virus” denotes uninfected controls. (D4: n=8 CON, n=7 TVA; D7: n=7 CON, n=7 TVA; D10: n=6 CON, n=7 TVA; n=4 no virus). **(E)** Representative flow cytometry plots (pre-gated on live CD4⁺ T cells) showing influenza-specific NP_311–325_ tetramer⁺ CD4⁺ T cells in lungs from CON and TVA groups at the indicated time points, with an uninfected control shown. Numbers indicate the percentage of tetramer⁺ cells within the plotted gate. **(F)** Frequency of CD8⁺ T cells among CD45⁺ lung cells at the indicated time points following PR8 infection; “No virus” denotes uninfected controls. (D4: n=8 CON, n=7 TVA; D7: n=7 CON, n=7 TVA; D10: n=6 CON, n=7 TVA; n=4 no virus). **(G)** Representative flow cytometry plots (pre-gated on live CD8⁺ T cells) showing influenza-specific PA_224–233_ tetramer⁺ CD8⁺ T cells in lungs from CON and TVA groups at the indicated time points, with an uninfected control shown. Numbers indicate the percentage of tetramer⁺ cells within the plotted gate. For (B) to (D) and (F), error bars indicate the mean ± SD; symbols indicate individual mice; data are representative of 2-3 independent experiments with 4-8 mice per group; statistical significance was determined by using two-way ANOVA and Holm-Sidak post-hoc tests.

**Fig. S8. TVA-milk effects on immune cell responses to lung viral infection. (A)** *Ex vivo* intracellular cytokine staining of lung T cells from PR8-infected neonatal pups in the CON and TVA groups. Frequencies of IFN-γ⁺ and T-bet⁺IFN-γ⁺ CD4⁺ T cells, TNF-α⁺ CD4⁺ T cells, and IFN-γ⁺ and TNF-α⁺ CD8⁺ T cells are shown (n=9) (**B**) Representative flow cytometry plots (pre-gated on live CD45⁺ cells) showing Foxp3⁺ CD4⁺ regulatory T cells (Tregs) in lungs from CON and TVA groups, with summary quantification of Treg frequencies among CD45⁺ cells in lung-infiltrating cells (*middle*) and splenocytes (*right*) (n=9). **(C-H)**, Flow cytometric quantification shows the effects of TVA-milk on lung-infiltrating neonatal B cell population (C), NK cell population (D), macrophage population (E), DC population (F), neutrophil population (G), and monocyte population (H) at the indicated days post-infection. “No virus” denotes uninfected controls. (D4: n=8 CON, n=7 TVA; D7: n=7 CON, n=7 TVA; D10: n=6 CON, n=7 TVA; n=4 no virus). **(I-J)** Effects of TVA-milk on lung (I) (n=5 each) and serum (J) (n=4 each) TNF-α level of 10-day-old mouse pups at 4/7/10 days post-viral infection. In (A) to (B), error bars indicate the mean ± SD; symbols indicate individual mice; data are representative of three independent experiments with nine mice per group. In (C) to (H), error bars indicate the mean ± SD; symbols indicate individual mice; data are representative of 2-3 independent experiments with 4-8 mice per group. For (I) to (J), error bars indicate the mean ± SEM; data are representative of two independent experiments with five mice per group. Statistical significance was determined by using an unpaired two-tailed Student’s t-test for (A) and (B), and two-way ANOVA and Holm-Sidak post-hoc tests for (C) to (J).

**Fig. S9.** **TVA-milk feeding for first-generation pups is sufficient to enhance early-life splenic immune development and improve neonatal influenza outcomes.** **(A)** Experimental design. Newly purchased 8-week-old C57BL/6J males and females were assigned to one of two TVA-exposure regimens: breeders were switched to **CON or TVA diet** **at the time of breeding setup**, or breeders were maintained on CON and switched to **TVA diet** **3 days prior to delivery (E17)**. First-generation pups (10–14 days old) were used for the neonatal influenza model and splenic immunophenotyping. The schematic figure was generated using BioRender. **(B)** Growth and immune phenotyping of 2-week-old pups. Body weight (*left*) and spleen weight (*middle*) are shown for pups from CON breeders and from breeders switched to TVA either at setup or at E17. *Right*, frequencies of major splenic immune populations (CD4⁺ T cells, CD8⁺ T cells, B cells, neutrophils, dendritic cells, and monocytes) among splenocytes. *Bottom*, differentiation status of splenic CD4⁺ and CD8⁺ T cells (naïve, central memory, and effector memory subsets). ***P* values** are shown. (CON: n=5; **at the time of breeding setup: n=5; 3 days prior to delivery: n=6**). **(C)** Neonatal influenza A challenge (10-day-old pups). *Left*, body weight immediately prior to viral challenge. *Middle*, clinical scores over time following infection. *Right*, Kaplan–Meier survival curves for pups from the indicated breeder-diet regimens (group sizes shown); ***P* values** are indicated. (CON: n=10; **at the time of breeding setup: n=13; 3 days prior to delivery: n=12**). In (B), violin plots depict the distribution with central tendency indicated (*left*), error bars indicate the mean ± SD; symbols indicate individual mice; data are representative of 2-3 independent experiments with ≥ 5 mice per group. For (C), error bars indicate the mean ± SD, symbols indicate individual mice (*left*); error bars indicate the mean ± SEM (*middle*); data are Kaplan–Meier overall survival curves (*right*); data are representative of three independent experiments with ≥ 10 mice per group. Statistical significance was determined by using one-way ANOVA and Tukey post-hoc tests for (B) and (C) *left*, two-way ANOVA and Holm-Sidak post-hoc tests for (C) *middle*, the log-rank (Mantel-Cox) test for (C) *right*,

**Fig. S10. Anti-viral function of TVA-milk requires a shift toward Th1-skewing neonatal immunity.** **(A)** Schematic depicting the experimental design. Lung tissues from pups with viral infection on CON- or TVA-milk (samples from 5 mice/group pooled) were analyzed by snRNA-seq and snATAC-seq. The figure was generated using BioRender. **(B)** Dot plot showing expression of canonical marker genes across lung cell clusters used for cell-type annotation. Dot size indicates the fraction of cells expressing each gene, and dot color denotes average (scaled) expression. **(C)** Relative *Gpr43* transcript level among cell clusters. Red bars represent TVA increased clusters, blue bars represent TVA decreased clusters, and black bars represent unchanged clusters. **(D)** SnRNA-seq–based comparison of ***Gpr43*** transcript levels between clusters whose abundance decreased versus increased in the TVA group (as defined in panel F); ***P* value** is shown. (n=10 decreased clusters, n=7 increased clusters). **(E)** UMAP plot displaying different subsets from snATAC-seq of lung tissue cells from pups with influenza A viral infection (numbers indicate total nuclei analyzed per group). Cells are colored by cluster identity, including Th1 cells and multiple CD8⁺ effector subclusters. **(F)** Cell proportion changes (TVA vs. CON) of different subsets in (D**),** point size indicates fold change. **(G)** Relative level of motif Z-score of TBX21_MA0690.1 in Th1 and CD8^+^ effector T cells between TVA and CON. **(H)** Relative level of motif Z-score of CREB1_MA0018.4 in Th1 and CD8^+^ effector T cells between TVA and CON. Dot size denotes the magnitude of motif Z-score change, and dot color indicates directionality as shown in the legend. For (D), violin plots show the distribution with summary statistics overlaid; symbols indicate cell clusters; statistical significance was determined by using an unpaired two-tailed Student’s t-test.

**Fig. S11**. **The protective effect of maternal TVA feeding against neonatal influenza requires T-bet and GPR43 on CD4⁺ T cells. (A)** Body weight of T-bet ^-/-^ pups in the TVA and CON group, prior to influenza A virus challenge (10-day-old) (n=4 CON, n=8 TVA). **(B)** Effects of TVA in breast milk on the clinical score of T-bet ^-/-^ mice with lung infection by influenza A virus at 10-day-old (n=4 CON, n=8 TVA). **(C)** Body weight of TCRα KO pups in the TVA and CON group, prior to influenza A virus challenge (10-day-old) (n=5 CON, n=8 TVA). **(D)** Effects of TVA in breast milk on the clinical score of TCRα KO mice with lung infection by influenza A virus at 10-day-old (n=5 CON, n=8 TVA). **(E)** Effects of TVA in breast milk on the survival rate of TCRα KO mice with lung infection by influenza A virus at 10-day-old (n=5 CON, n=8 TVA). **(F)** Body weight of CD4 KO pups in the TVA and CON group, prior to influenza A virus challenge (10-day-old) (n=13 CON, n=15 TVA). **(G)** Effects of TVA in breast milk on the clinical score of CD4 KO mice with lung infection by influenza A virus at 10-day-old (n=13 CON, n=15 TVA). **(H)** Effects of TVA in breast milk on the survival rate of CD4 KO mice with lung infection by influenza A virus at 10-day-old (n=13 CON, n=15 TVA). **(I)** Body weight of *Gpr43*^-/-^ pups in the TVA and CON group, prior to influenza A virus challenge (10-day-old) (n=6 CON, n=6 TVA). **(J)** Effects of TVA in breast milk on the clinical score of *Gpr43*^-/-^ mice with lung infection by influenza A virus at 10-day-old (n=6 CON, n=6 TVA). **(K)** Effects of TVA in breast milk on the survival rate of *Gpr43*^-/-^ mice with lung infection by influenza A virus at 10-day-old (n=6 CON, n=6 TVA). **(L)** Body weight of *Gpr43*^fl/fl^CD4^cre^ pups in the TVA and CON group, prior to influenza A virus challenge (10-day-old) and effects of TVA in breast milk on the clinical score of *Gpr43*^fl/fl^CD4^cre^ mice with lung infection by influenza A virus at 10-day-old (n=13 CON, n=11 TVA) (*left*); Body weight and clinical score of littermate control in the TVA and CON group (n=19 CON, n=10 TVA) (*right*). For (A), (C), (F), and (I), error bars indicate the mean ± SD; symbols indicate individual mice. For (B), (D), (G), and (J), error bars indicate the mean ± SEM. For (E), (H), and (K), data are Kaplan–Meier overall survival curves. Data are representative of 3-5 independent experiments with ≥ 4 mice per group (A to K). For (L), error bars indicate the mean ± SD, symbols indicate individual mice (body weight data) or mean ± SEM (clinical score data). Data are representative of four independent experiments with ≥ 10 mice per group. Statistical significance was determined by using an unpaired two-tailed Student’s t-test for (A), (C), (F), (I), and body weight data of (L), two-way ANOVA and Holm-Sidak post-hoc tests for (B), (D), (G), (J), and clinical score data of (L), the log-rank (Mantel-Cox) test for (E), (H), and (K).

.

**Fig. S12. Competitive adoptive transfer for tracking TVA- versus CON–milk–programmed neonatal naïve CD4⁺ T cells *in vivo*.** (**A**) Experimental design. Neonatal naïve CD4⁺ T cells were isolated from CD45.1 pups nursed by CON-fed dams (CON donors) and from CD45.1/2 pups nursed by TVA-fed dams (TVA donors) at postnatal day 10–15. Donor cells were mixed at a 1:1 ratio (validated in panel B) and adoptively transferred (*i.v.*; 4×10⁶ total mixed cells per mouse, day 0) into CD45.2 recipient mice. Endogenous recipient CD4⁺ T cells were depleted by anti-CD4 (GK1.5, i.p.) at 2 weeks of age (day −14), and adoptive transfer was performed when recipients were 4 weeks old (n = 13). Recipients were challenged with influenza A virus 1 day post-transfer (day 1) and tissues were harvested on day 8 for analysis. The figure was generated using BioRender**.** (**B**) Flow cytometry gating strategy confirming the 1:1 donor mix prior to transfer. Lymphocytes and singlets were gated, followed by CD4⁺ cells, and donor populations were distinguished by congenic markers (CD45.1 vs CD45.1/2). (**C**) Representative gating strategy for quantifying donor contributions in recipient spleen and lung-infiltrating cells after infection. Donor cells were identified within live lymphocytes/singlets and CD4⁺ gates; in lungs, analysis focused on donor-derived CD4⁺T-bet^hi^ cells before assessing the relative frequencies of CD45.1/2 (TVA-derived) versus CD45.1 (CON-derived) donor cells. (**D**) Summary of the competitive advantage of TVA-derived donors, shown as the ratio of CD45.1/2 to CD45.1 donor cells in recipient spleen (CD4⁺) and lung (CD4⁺T-bet^hi^) compartments; *P* value is shown; each pair of points connected by a line represents an individual recipient mouse (paired analysis); blue circles denote spleen and red circles denote lung. Statistical significance was determined by using a paired two-tailed Student’s t-test.

**Fig. S13**. **TVA-milk–mediated modulation of neonatal mouse immune responses to Salmonella Typhimurium challenge.** (**A**) Body weight of 2-week-old pups from CON- and TVA-fed dams measured immediately prior to oral *S. Typhimurium* challenge and on day 3 post-challenge (n=5 CON, n=8 TVA); P values are shown. (**B–C**) Flow cytometric analysis of splenic T cell responses on day 3 post-challenge. Shown are the frequencies of CD4⁺ T cells among CD45⁺ cells and IFN-γ⁺ cells among CD4⁺ T cells (B), and the frequencies of CD8⁺ T cells among CD45⁺ cells and IFN-γ⁺ cells among CD8⁺ T cells (C) (n=5 CON, n=8 TVA). (**D**) Frequencies of major splenic immune populations among CD45⁺ cells on day 3 post-challenge, including B cells, NK cells, macrophages, dendritic cells (DCs), neutrophils, and monocytes (n=5 CON, n=8 TVA). For (A) to (D), error bars indicate the mean ± SD; symbols indicate individual mice; data are representative of two independent experiments with ≥ 12 mice per group. Statistical significance was determined by using an unpaired two-tailed Student’s t-test for (A) to (D).

**Fig. S14. Maternal TVA increases Th1-associated cytokine signals in neonatal mouse serum and directly promotes Th1 differentiation of human cord blood naïve CD4⁺ T cells.** **(A)** Experimental design and serum cytokine profiling. Two-week-old pups nursed by dams fed CON or TVA diets were analyzed using the Proteome Profiler Mouse Serum Cytokine Array (*left*). The figure was generated using BioRender**.** Volcano-style plot showing relative signal intensity (TVA/CON) versus −log₁₀(P) for detected factors; selected cytokines/chemokines are annotated, and the dashed line indicates the nominal ***P* = 0.05** threshold, top candidates of positive (red circles) and negative (blue circles) are shown (*right*) (n=4 each). (**B**) Representative flow cytometry plots from human umbilical cord blood sample 1 naïve CD4⁺ T cells treated with vehicle (CON) or TVA (20 µM or 100 µM) and then cultured under Th1-polarizing conditions for 48 h or 72 h. Gates show T-bet⁺ cells and T-bet⁺IFN-γ⁺ cells within CD4⁺ T cells, with frequencies indicated. (**C**) Quantification of Th1 differentiation across three independent cord blood donors. Frequencies of T-bet⁺ and T-bet⁺IFN-γ⁺ cells within CD4⁺ T cells are shown after Th1 polarization for 48 h or 72 h, as indicated (5 technical replicates). In (C), error bars indicate the mean ± SD; symbols indicate technical replicates of each human sample; each human sample is representative of one independent experiment (3 human samples in total). Statistical significance was determined by using an unpaired two-tailed Student’s t-test for (A), one-way ANOVA and Tukey post-hoc tests for (C).

**Fig. S15. Effects of postnatal and prenatal exposure to TVA on immune response to lung viral infection. (A)** Serum TVA concentrations in 10-day-old pups following cross-fostering between CON- and TVA-fed dams (n=5). **(B)** Body weight of pups in each group of the mouse-fostering experiment, prior to influenza A virus challenge (10-day-old) (n numbers are indicated in the figure). **(C)** Effects of TVA exposure in breast milk or during pregnancy on the clinical score of mice with lung infection by influenza A virus at 10-day-old. Brackets indicate statistical comparisons among the four fostering conditions (ns, not significant; P<0.05 as indicated) (n= 10 CON to CON, n=8 CON to TVA, n=5 TVA to CON, n=6 TVA to TVA)**. (D)** TVA-milk but not during pregnancy enhanced anti-viral immunity of pups with reduced viral loads in lungs assessed by decreased influenza virus *PR8 NS1* mRNA levels on Day 7 post-infection to pups at 10-day-old (n= 7 CON to CON, n=7 CON to TVA, n=6 TVA to CON, n=5 TVA to TVA). **(E)** Effects of TVA exposure in breast milk or during pregnancy on lung-infiltrating neonatal CD4^+^ T cell percentage, %CD11a^+^CD49d^+^ of CD4^+^, %CD44^+^ of CD4^+^ cells (n= 7 CON to CON, n=7 CON to TVA, n=6 TVA to CON, n=5 TVA to TVA). **(F)** Effects of TVA exposure in breast milk or during pregnancy on lung-infiltrating neonatal CD8^+^ T cell population (*left*), activation (CD69, *middle*), and virus-recognizing activity (PA_224-233_-specific, *right*) (n= 7 CON to CON, n=7 CON to TVA, n=6 TVA to CON, n=5 TVA to TVA). **(G)** Effects of TVA exposure in breast milk or during pregnancy on lung-infiltrating neonatal NK cell population, B cell population, and macrophage population (n= 7 CON to CON, n=7 CON to TVA, n=6 TVA to CON, n=5 TVA to TVA). **(H)** Effects of TVA exposure in breast milk or during pregnancy on lung-infiltrating neonatal DC population, neutrophil population, and monocyte population (n= 7 CON to CON, n=7 CON to TVA, n=6 TVA to CON, n=5 TVA to TVA). For (A), (B), and (D) to (H), error bars indicate the mean ± SD; symbols indicate individual mice; data are representative of three independent experiments with ≥ 5 mice per group. For (C), error bars indicate the mean ± SEM; data are representative of three independent experiments with ≥ 5 mice per group. Statistical significance was determined by using one-way ANOVA and Tukey post-hoc tests for (B), and (D) to (H)**,** two-way ANOVA and Holm-Sidak post-hoc tests for (C).

**Fig. S16**. **TVA-driven immune imprinting is established during early life and is not efficiently induced by TVA exposure in adult naïve CD4⁺ T cells.**

**(A)** Effects of having TVA-enriched breast milk before weaning (0-3 weeks old) on the body weight prior to influenza A virus challenge at 6-10 weeks old (n=9 CON to CON, n=7 TVA to CON). **(B)** Effects of having TVA-enriched breast milk before weaning (0-3 weeks old) on the clinical score with infection by influenza A virus at 6-10 weeks old (n=9 CON to CON, n=7 TVA to CON), asterisks denote statistically significant differences between groups at the indicated time points. **(C)** Representative chemiluminescent EMSA showing CTCF activity difference between naïve CD4⁺ T cells from adult mice that were on TVA-milk or control milk during the pre-weaning period (0–3 weeks of age). Positions of the shifted CTCF–DNA complex and free probe are indicated. **(D)** Effects of having TVA-enriched breast milk before weaning (0-3 weeks old) on the body weight prior to influenza A virus challenge at 8-12 weeks old (n=10 CON to CON, n=12 TVA to CON). **(E)** Effects of having TVA-enriched breast milk before weaning (0-3 weeks old) on the clinical score with infection by influenza A virus at 8-12 weeks old (n=10 CON to CON, n=12 TVA to CON), asterisks denote statistically significant differences between groups at the indicated time points. (**F**) Effects of having TVA-enriched breast milk before weaning (0-3 weeks old) on the survival rate with infection by influenza A virus at 8-12 weeks old (n=10 CON to CON, n=12 TVA to CON). **(G)** Effects of the TVA diet during 5–8 weeks of age on the body weight prior to influenza A virus challenge at 10-12 weeks old (n=13 CON to CON, n=10 TVA to CON). **(H)** Effects of the TVA diet during 5–8 weeks of age on the clinical score with infection by influenza A virus at 10-12 weeks old (n=13 CON to CON, n=10 TVA to CON). **(I)** Experimental scheme for *in vitro* priming of naïve CD4⁺ T cells from neonatal (10–14 days old) or adult (8–12 weeks old) mice with TVA or DMSO (plus IL-7), followed by anti-CD3/CD28 activation and culture under Th1/Th2/Th17-polarizing conditions. The figure was generated using BioRender. **(J)** Quantification of Th lineage polarization following the *in vitro* treatment shown in (F). TVA increases **Th1 polarization** (T-bet⁺IFN-γ⁺) in neonatal naïve CD4⁺ T cells but shows minimal effect on adult naïve CD4⁺ T cells, while **Th2** (GATA3⁺IL-4⁺) and **Th17** (RORγt⁺IL-17⁺) polarization rates are not significantly altered under the indicated conditions. In (A), (D), and (G), error bars indicate the mean ± SD; symbols indicate individual mice; data are representative of 3-4 independent experiments with ≥ 7 mice per group. For (B), (E), and (H), error bars indicate the mean ± SEM; data are representative of 3-4 independent experiments with ≥ 7 mice per group. For (J), error bars indicate the mean ± SD; symbols indicate individual mice; data are representative of two independent experiments with four mice per group. Statistical significance was determined by using an unpaired two-tailed Student’s t-test for (A), (D), and (G), two-way ANOVA and Holm-Sidak post-hoc tests for (B), (E), (H), and (J), and the log-rank (Mantel-Cox) test for (F).

**Fig. S17**. **Working model.** The figure was generated using BioRender.

Data S1. (separate file): Spleen cell Gene Promoter Sum changes by cluster

This table shows the changes in chromatin accessibility (Gene Promoter Sum) of splenic cells of pups on TVA- vs. CON- milk at the age of 2 weeks.

**Data S2. (separate file): Spleen cell motif changes by cluster**

This table shows the changes in the motif of splenic cells of pups on TVA- vs. CON- milk at the age of 2 weeks.

**Data S3.** **(separate file**)**: Spleen cell cluster marker genes**

This table presents the marker genes that define spleen cell clusters in mice receiving TVA- or CON-milk at 2 weeks of age.

**Data S4. (separate file): Mouse milk lipid profile**

This table lists the levels of different fatty acids in the milk of mouse dams fed either a TVA or CON diet, with 4 samples per group.

**Data S5.** **(separate file): Lung cell gene expression changes by cluster**

This table shows changes in gene expression in lung cells (by cluster) from pups fed TVA- versus CON-milk, measured 7 days after influenza A virus challenge at 10 days of age.

**Data S6. (separate file): Lung cell motif changes by cluster**

This table shows changes in motif in lung cells (by cluster) from pups fed TVA- versus CON-milk, measured 7 days after influenza A virus challenge at 10 days of age.

**Data S7. (separate file): Lung cell cluster marker genes**

This table presents the marker genes that define lung cell clusters from pups fed TVA- versus CON-milk, measured 7 days after influenza A virus challenge at 10 days of age.

**Data S8. (separate file): 2-week-old mice serum cytokines**

This table lists the levels of different serum cytokines in 2-week-old mice fed TVA- versus CON-milk, with 4 samples per group.

**Data S9. (separate file): Infant blood sport card samples for cytokine level checking**

This table lists the milk source and postmenstrual age at sample collection for infant dried blood spot card samples used for cytokine measurement.

**Data S10. (separate file): Preterm infants’ clinical and milk information: Human milk samples for fatty acid profiling**

This table lists the milk source, BPD status, gestational age, and postmenstrual age at sample collection for preterm infants whose paired breast milk samples were used for fatty acid profiling.

**Data S11. (separate file): Milk FA profiling summary for BPD**

This table presents the fatty acid profiling results of paired breast milk samples, shown as fold changes in milk fatty acid levels between the no-BPD and BPD groups, which were defined based on whether the preterm infants developed BPD.

**Data S12. (separate file): Preterm infants’ clinical and milk information: Infant blood spot card samples for TVA level checking**

This table summarizes the milk source, BPD status, gestational age, and postmenstrual age at the time of sample collection for dried blood spot card samples from preterm infants analyzed for TVA levels.

**Data S13. (separate file): Antibodies**

This table lists all antibodies used in this study.
